# From nerve to bone: a tale of autonomic nervous system, bone cells, and beyond

**DOI:** 10.1016/j.mmr.2026.100051

**Published:** 2026-06-29

**Authors:** Wei-Jun Huang, Yong-Kang Hu, Heng-Zhen Li, Wen-Hao Lu, Guang Yang, Bing-Zhou Ji, Wen-Feng Xiao, Yu-Sheng Li

**Affiliations:** a Department of Orthopedics, Xiangya Hospital, Central South University, Changsha 410008, China; b National Clinical Research Center for Geriatric Disorders, Xiangya Hospital, Central South University, Changsha 410008, China; c Xiangya School of Medicine Central South University, Changsha 410083, China

**Keywords:** Autonomic nervous system (ANS), Neurotransmitters, Bone tissue cells, Bone metabolism, Bone-related diseases, Therapeutic strategies

## Abstract

Bone is a multifunctional organ essential for structural support, protection, hematopoiesis, and endocrine regulation. Emerging research increasingly highlights the pivotal role of the nervous system in bone metabolism, repair, and musculoskeletal disease progression, particularly the autonomic nervous system (ANS), which precisely regulates bone tissue cells via sympathetic and parasympathetic pathways. The ANS regulates the activity of mesenchymal stem cells (MSCs), osteoblasts, osteoclasts, chondrocytes, and nucleus pulposus cells (NPCs) via neurotransmitters such as norepinephrine (NE), acetylcholine (ACh), neuropeptide Y (NPY), and vasoactive intestinal peptide (VIP), thereby modulating core pathological processes in bone-related disorders including osteoporosis (OP), osteoarthritis (OA), intervertebral disc degeneration (IVDD), and traumatic fractures. Targeted neuromodulation is emerging as a promising therapeutic strategy for musculoskeletal disorders. This review aims to summarize the regulatory roles of the ANS in bone cell metabolism and bone-related pathologies and to evaluate current ANS-bone axis-based interventions to promote skeletal health and alleviate the burden of musculoskeletal diseases.

## 1 Background

Bone serves as a vital organ in mammals, providing not only structural support and protection for internal organs but also participating in key physiological processes such as hematopoiesis, mineral storage, and endocrine regulation [1], [2]. To sustain these diverse functions, the nervous system plays an essential role in regulating bone metabolism, maintaining homeostasis, and promoting tissue repair [3]. Nerve fibers are extensively and intricately distributed in the skeleton, particularly in the periosteum, mineralized bone, and bone marrow. They not only mediate mechanical and pain sensation but also serve critical functions in maintaining skeletal integrity and facilitating bone repair [4], [5], [6]. In addition to regulating bone tissue cells, the nervous system is also deeply involved in the development and progression of various skeletal disorders, such as osteoporosis (OP) and osteoarthritis (OA) [7]. Therefore, the nervous system is essential for maintaining skeletal physiology.

The autonomic nervous system (ANS) consists of the sympathetic nervous system (SNS) and the parasympathetic nervous system (PSNS), which often exhibit antagonistic physiological effects [8]. The SNS primarily mediates stress responses by releasing norepinephrine (NE), which promotes bone resorption and influences cellular metabolism within the joints [9]. In contrast, the PSNS mainly secretes acetylcholine (ACh) to regulate inflammation and repair processes in bone and joint tissues [10]. Meanwhile, enhanced parasympathetic activity can reduce the levels of inflammatory mediators in synovial fluid, thereby alleviating joint pain and swelling [11]. Furthermore, sympathetic nerve fiber-derived neurotransmitters such as vasoactive intestinal peptide (VIP) are pivotal in intervertebral disc degeneration (IVDD), mitigating inflammation and degeneration of disc cells via the fibroblast growth factor (FGF) 18/fibroblast growth factor receptor (FGFR) 2 signaling pathway [12]. Together, these findings highlight the essential role of the ANS in skeletal homeostasis, repair, and the pathogenesis as well as management of musculoskeletal diseases.

**Fig. 1:**
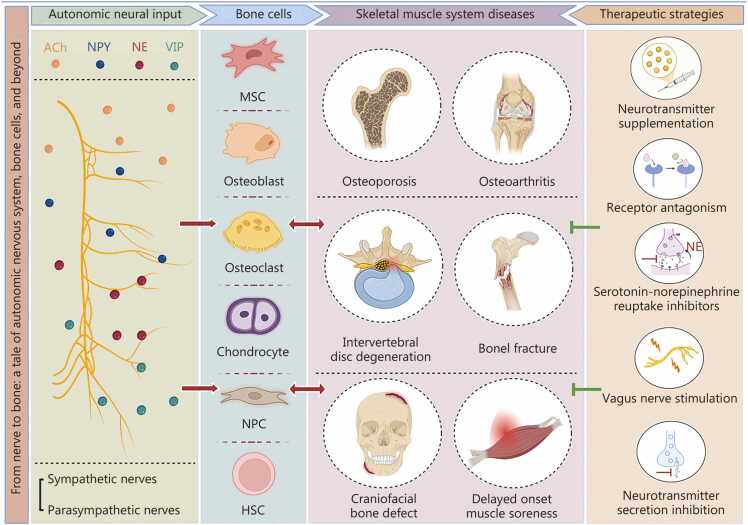
Overview of autonomic nerve input patterns, the interactions among autonomic nerves, bone cells, and skeletomuscular pathological conditions, and the corresponding therapeutic strategies. ACh. Acetylcholine; NPY. Neuropeptide Y; NE. Norepinephrine; VIP. Vasoactive intestinal peptide; NPC. Nucleus pulposus cell; HSC. Hematopoietic stem cell; MSC. Mesenchymal stem cell.

The ANS regulates not only bone homeostasis and repair at the systemic level, but also precisely modulates the differentiation of mesenchymal stem cells (MSCs) [13], [14], the activity of osteoblasts [15] and osteoclasts [16], [17], the fate of chondrocytes [18] and nucleus pulposus cells (NPCs) [19], [20], and the maintenance of hematopoietic stem cell (HSC) niches in the bone marrow [21], underscoring its multilayered regulatory capacity. For instance, the cholinergic activity of the PSNS promotes osteoblast proliferation and induces osteoclast apoptosis, which supports bone repair and remodeling [22]. This review aims to systematically analyze the complex interactions between the ANS and the skeletal system, further elucidate the potential roles of the nervous system in bone metabolism and bone-related diseases, and explore its prospects as a therapeutic target in clinical applications (Fig. 1).

## 2 Autonomic nervous system in bone: neurotransmitters and receptors

**Fig. 2:**
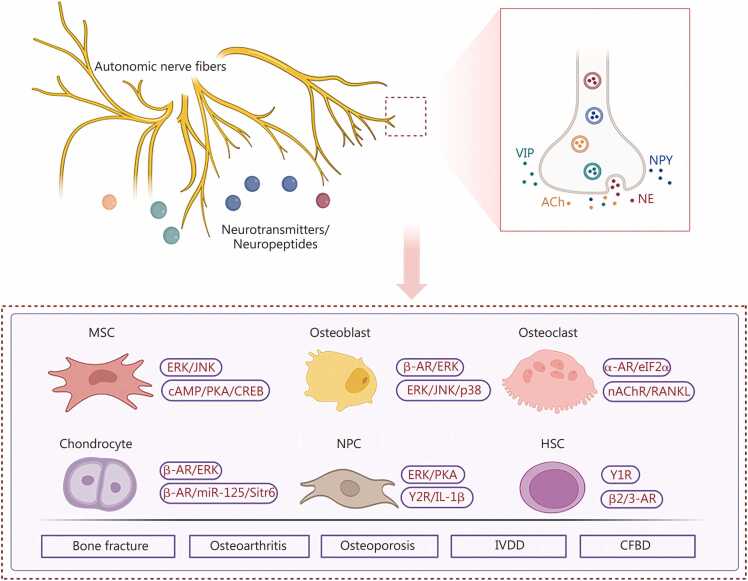
Overview of the essential regulatory functions of autonomic nerves and their associated neurotransmitters and neuropeptides in modulating the proliferation, differentiation, and activity of mesenchymal stem cells (MSCs), osteoblasts, osteoclasts, chondrocytes, hematopoietic stem cells (HSCs), and nucleus pulposus cells (NPCs) in the pathophysiology of several key bone-related disorders. VIP. Vasoactive intestinal peptide; NPY. Neuropeptide Y; ACh. Acetylcholine; NE. Norepinephrine; ERK. Extracellular signal-regulated kinase; JNK. c-Jun N-terminal kinase; cAMP. Cyclic adenosine monophosphate; PKA. Protein kinase A; CREB. cAMP response element-binding protein; β-AR. Beta-adrenergic receptor; α-AR. Alpha-adrenergic receptor; eIF2α. Eukaryotic translation initiation factor 2 alpha; nAChR. Nicotinic acetylcholine receptor; RANKL. Receptor activator of nuclear factor kappa-B ligand; Y2R. Neuropeptide Y receptor type 2; IL. Interleukin; IVDD. Intervertebral disc degeneration; CFBD. Cortical fenestration bone defect.

The nervous system consists of the central nervous system (CNS), which includes the brain and spinal cord, and the peripheral nervous system, which comprises the cranial and spinal nerves. The peripheral nervous system is further divided into the somatic nervous system and ANS, also referred to as the vegetative nervous system. The sympathetic and parasympathetic nerves of the ANS are extensively and intricately distributed throughout the cardiac muscle, smooth muscle, and glands [23], [24]. Beyond classical neurotransmitters such as NE and ACh, bioactive factors including neuropeptide Y (NPY), VIP, dopamine (DA), and leptin, as well as their corresponding receptors, specifically modulate ANS-mediated bone metabolism (Fig. 2). Evidence from animal studies demonstrated that autonomic nerve fibers innervate the periosteum during embryonic development and later extend into the metaphyseal and epiphyseal regions of long bones [5], [25]. Specifically, sympathetic nerve fibers are particularly densely distributed in bone tissue, especially within the periosteum [5].

### 2.1 Sympathetic regulation via norepinephrine-adrenergic receptor signaling

NE is primarily synthesized and released by the sympathetic nerves, with a small amount secreted by chromaffin cells in the adrenal medulla. It serves as the main neurotransmitter of sympathetic signaling [5]. The synaptic transmission of NE mainly comprises a series of well-organized steps, wherein NE is synthesized in neurons, packaged into vesicles, released into the synaptic cleft, reabsorbed by transporters, and ultimately degraded by enzymes [24]. The synthesis of NE begins with tyrosine, which is converted to L-3,4-dihydroxyphenylalanine (L-DOPA) by tyrosine hydroxylase. L-DOPA is then converted to DA by L-aromatic amino acid decarboxylase. DA is subsequently transported into synaptic vesicles by the vesicular monoamine transporter, where it is converted to NE by DA β-hydroxylase [26].

Adrenergic receptors (ARs) primarily mediate their functions through binding to NE and epinephrine [27]. Molecular cloning has revealed that the family of alpha-adrenergic receptors (α-ARs) comprises two distinct subtypes, α1-AR and α2-AR, while the family of beta-adrenergic receptors (β-ARs) encompasses β1-AR, β2-AR, and β3-AR subtypes. All AR subtypes are members of the G protein-coupled receptor (GPCR) superfamily [28].

### 2.2 Parasympathetic modulation via cholinergic signaling

ACh functions as the principal neurotransmitter of the PSNS, governing involuntary physiological processes including heart rate, gastrointestinal motility, smooth muscle tone, and exocrine gland secretion [8]. ACh-mediated neurotransmission is orchestrated by a sequence of tightly integrated steps: ACh synthesis, vesicular packaging, storage within synaptic vesicles, exocytotic release into the synaptic cleft, reuptake via presynaptic transporters, and enzymatic degradation by acetylcholinesterase [24].

Cholinergic receptors are divided into two distinct categories according to their responsiveness to the specific agonists nicotine and muscarine: nicotinic acetylcholine receptors (nAChRs) and muscarinic acetylcholine receptors (mAChRs). Among them, nicotinic receptors include the neuronal type (N1), which is in the CNS and autonomic ganglia, and the muscle type (N2), which is found at neuromuscular junctions. Muscarinic receptors are chiefly localized in autonomic effector organs and comprise five subtypes (M1–M5), all of which function as GPCRs [29], [30].

### 2.3 Roles of other neuropeptides, related neuromodulators, and their receptors

#### 2.3.1 Neuropeptide Y

NPY is a highly conserved 36-amino acid peptide, predominantly found in the arcuate nucleus of the hypothalamus within the CNS. It can also be released by the adrenal medulla and the sympathetic nerves in response to chronic stress [31]. NPY receptors include five subtypes: Y1Rs, Y2Rs, Y4Rs, Y5Rs, and Y6Rs [32], all of which are GPCRs. They are highly expressed in various brain regions and the ANS. Among them, Y1Rs, Y2Rs, and Y6Rs are involved to some extent in the regulation of bone homeostasis [31], [33], [34]. Previous studies demonstrated the osteoprotective effects of Y2R ablation, revealing that global knockout increased trabecular bone formation [34], [35], [36], [37], whereas hypothalamus-specific deletion prevented sex hormone deficiency-induced bone loss [38]. Collectively, Y2Rs function as critical negative regulators of bone mass, indicating that the targeted inhibition of central or peripheral Y2R signaling may offer a promising strategy to mitigate pathological bone loss induced by chronic stress.

#### 2.3.2 Vasoactive intestinal peptide

VIP was originally discovered to be expressed in the small intestine and lungs. VIP is widely distributed throughout the SNS [39]. In the PSNS, VIP is typically co-released with ACh at parasympathetic nerve terminals [8]. The VIP released from these fibers plays a key role in regulating bone metabolism, bone remodeling, and maintaining bone homeostasis [40], [41]. VIP mainly exerts biological effects through two GPCR-B: vasoactive intestinal peptide receptor (VPAC)1 and VPAC2 [42], [43]. Furthermore, VPAC1 and VPAC2 are expressed on both osteoblasts and osteoclasts during cranial bone differentiation [44], [45]. Upon specific receptor engagement, VIP orchestrates the functional coupling of these bone cells, thereby preserving skeletal integrity.

#### 2.3.3 Dopamine

DA is a neuropeptide widely distributed in the CNS and peripheral tissues. In the peripheral tissues, DA, as a precursor of NE and epinephrine, is abundantly stored in sympathetic nerves and can directly modulate skeletal cells to regulate bone metabolism [16]. A previous study showed that mice with DA transporter deficiency exhibit low bone mass, highlighting an essential contribution of the dopaminergic pathway in regulating bone remodeling [46]. DA deficiency or excess is linked to neuropsychiatric disorders such as Parkinson’s disease and schizophrenia. Patients with these conditions often have a higher risk of osteoporotic fractures than healthy individuals, suggesting that DA levels may influence bone mass [47]. DA exerts its biological effects through five distinct GPCRs, namely D1–D5 [48], [49], [50]. These receptors are found in various cell types, such as bone marrow mesenchymal stem cells (BMSCs), osteoblasts, and osteoclasts, and participate extensively in modulating the skeletal microenvironment [16], [51], [52].

#### 2.3.4 Leptin

Leptin is a cytokine-like peptide hormone secreted by adipose tissue, essential for the regulation of food intake and energy metabolism [53], [54]. It has been identified that leptin receptors are expressed in peripheral tissues such as skeletal muscle and bone, as well as in various skeletal cell types [55], [56]. However, the role of leptin in regulating bone homeostasis remains a subject of debate. To address this controversy, we propose a unifying “metabolism-dependent central-peripheral dual-regulation model”. Within this framework, the dominant regulatory mechanism shifts dynamically depending on central leptin sensitivity. Within this framework, when central leptin sensitivity is preserved under physiological conditions, regulatory control is predominantly exerted through central pathways. In this state, elevated central leptin levels amplify sympathetic tone, which suppresses osteoblast proliferation and stimulates osteoclast activity primarily via β2-ARs, ultimately accelerating trabecular bone loss [57], [58], [59], [60]. Conversely, the peripheral circuit acts as the primary mediator under metabolic disorders such as obesity and metabolic syndrome. Specifically, obesity-induced central leptin resistance attenuates central inhibitory signaling. Accordingly, circulating leptin directly activates long-form receptors (LepRb) on bone cells, eliciting cellular responses that counteract central regulation. This local interaction promotes cortical bone apposition and increases total bone mass [55], [56], [61]. To validate this model, future studies should investigate osteoblast-specific *Lepr* knockout mice (such as Osx-Cre; Lepr^fl/fl^) across different metabolic conditions. Comparing these mutants with wild-type controls under normal or high-fat dietary conditions effectively verifies the distinct peripheral and central mechanisms. Under obese conditions, wild-type mice are expected to exhibit peripheral leptin-driven bone formation, whereas the knockout models lack this response. This strategy would confirm that the peripheral circuit dominates the central axis only after central resistance develops.

#### 2.3.5 Endocannabinoids

The endocannabinoid system (ECS) comprises endogenous cannabinoids, their receptors [cannabinoid receptor type 1 (CB1) and cannabinoid receptor type 2 (CB2)], and a set of downstream enzymes [62], [63]. Notably, CB1 is found at peripheral sympathetic nerve terminals associated with bone [64], with minimal expression on the surface of BMSCs, osteoblasts, osteoclasts, and osteocytes. In contrast, CB2 is highly expressed in osteoblasts, osteoclasts, and osteocytes [65], [66]. The ECS can exert a critical influence on regulating bone remodeling and bone quality by modulating the SNS [62], [67], [68]. Researchers found that endogenous cannabinoid 2-arachidonoylglycerol (2-AG) produced by osteoblasts binds to presynaptic CB1 in sympathetic neurons, thereby inhibiting NE release. This reduced the NE-induced suppression of bone formation mediated by β2-ARs on osteoblasts [65], [67], [69]. In summary, the ECS is essential for skeletal health.

## 3 Autonomic nervous system and neuropeptide-mediated regulation of bone tissue cells

### 3.1 Fate of mesenchymal stem cells

MSCs are multipotent progenitor cells derived from the mesoderm and characterized by a fibroblast-like morphology. They can differentiate into osteoblasts and chondrocytes, thereby contributing to skeletal homeostasis and participating in the pathogenesis of age-related diseases as well as in cell-based therapeutic strategies [70]. Bone marrow stromal MSCs have been extensively characterized and are regarded as a principal MSC source because of their abundance and relatively stable differentiation capacity [71]. With the growing interest in neuro-skeletal interactions, recent studies have increasingly highlighted the regulatory role of the ANS in determining MSC fate, particularly through sympathetic signaling [13], [34], [72]. Notably, the evidence suggests that the SNS not only directly regulates BMSC differentiation but also modulates the bone marrow microenvironment to indirectly influence BMSC-HSC interactions [13].

The ANS finely modulates MSCs through various neurotransmitters and regulatory factors. Within sympathetic signaling networks, NE exerts divergent, context-dependent effects on MSC biology. For instance, high concentrations of NE (10^–6^–10^–5^ mol/L) markedly suppressed human BMSC proliferation by activating the β2-AR-mediated extracellular signal-regulated kinase (ERK)1/2-protein kinase A (PKA) pathway [73]. However, this inhibitory effect occurred under sustained, supraphysiological NE exposure *in vitro*, potentially amplifying β2-AR signaling beyond its physiological range. Thus, this robust response likely represents an exaggerated form of baseline β2-AR regulation rather than a direct reflection of normal sympathetic tone. In contrast, under severe metabolic stress, particularly glucotoxicity, NE exerted a paradoxical cytoprotective effect on murine MSCs. Mechanistically, NE signaling activated the protein kinase B (Akt)/B-cell lymphoma 2 (Bcl-2) axis via α-ARs, downregulating Bcl-2-associated X protein (Bax) and cleaved caspase-3 while limiting reactive oxygen species (ROS) accumulation to attenuate apoptosis [74]. Crucially, this α-AR-mediated survival response emerges specifically under pathological stress, suggesting a microenvironment-dependent shift in receptor engagement. Collectively, these findings imply a physiological “receptor shift” in the sympathetic regulation of MSCs. Under basal conditions, β2-AR signaling appears to exert a tonic restraint on osteogenesis, consistent with *in vivo* evidence from rabbit models where β2-AR blockade significantly upregulated osteogenic markers [75]. Conversely, during metabolic or oxidative stress, α-AR pathways may dominate to promote cell survival as a compensatory adaptation. Nevertheless, extrapolating extreme *in vitro* paradigms directly to physiological bone remodeling warrants caution. Rigorous validation necessitates graded, physiologically relevant NE exposure models in genetically tractable mice, combined with MSC-lineage-specific adrenergic receptor beta 2 (*Adrb2*) deletion and subtype-selective α-AR modulation to delineate nerve-derived from cell-autonomous effects. Integrating this framework with lineage tracing and dynamic histomorphometry will ultimately determine whether a context-dependent shift in receptor dominance truly occurs *in vivo*.

In concert with the sympathetic axis, cholinergic signaling dictates MSC dynamics and lineage commitment. *Ex vivo* analyses of human trauma-derived MSCs revealed that AChR levels dynamically shift, exhibiting upregulation during osteogenesis and downregulation during adipogenesis [76]. However, these *ex vivo* findings primarily establish correlative, rather than causal, relationships. For instance, MSCs derived from trauma tissues are exposed to severe inflammation, which can easily alter the basal receptor expression of the cells. Additionally, *in vitro* experiments in rat BMSCs indicated that native ACh promoted cell migration via the m1AChR-mediated Ca²⁺/protein kinase C (PKC)/ERK1/2 signaling pathway [77]. Moreover, treating human MSCs with the nAChR agonist nicotine elicited a biphasic response: low doses (10⁻⁷ mol/L) promoted Ca²⁺ influx and cell migration, whereas higher doses (10⁻⁵ mol/L) paradoxically induced apoptosis and impaired directed chemotaxis [78]. Unlike natural ACh, nicotine is poorly degraded by the enzyme, which prolongs cholinergic channel opening, leading to toxic calcium overload [78]. Consequently, this *in vitro* model may not fully represent the brief, natural cholinergic signaling that occurs *in vivo*.

In addition, ANS-mediated secretion of osteocyte-derived NPY suppressed BMSC osteogenesis while promoting adipogenesis, leading to a bone-fat metabolic imbalance. Notably, this negative regulation was reversed in an osteocyte-specific NPY^fl/fl^ mouse model [79]. Conversely, in a rat femoral fracture model, melatonin intervention accelerated fracture healing by upregulating NPY and Y1Rs, which significantly enhanced MSC proliferation and migration. This pro-osteogenic effect was blunted by the Y1R antagonist BIBP3226 [80]. Such divergent effects of NPY on BMSC fate determination are likely due to a distinct microenvironment. Within physiological homeostasis, osteocyte-derived NPY may act as a paracrine inhibitor to restrict basal bone formation. In contrast, during acute trauma such as fractures, NPY-signaling may be dynamically reprogrammed into a pro-regenerative stimulus, mobilizing rapid BMSC expansion and tissue repair.

In cultured human BMSCs, VIP upregulated early osteogenic markers and facilitated cytoskeletal remodeling required for bone matrix formation [34]. In a rat cranial defect model, VIP further enhanced bone regeneration and local angiogenesis. Mechanistically, these effects were associated with activation of the Wnt/β-catenin pathway in BMSCs, leading to augmented osteogenic differentiation and increased vascular endothelial growth factor (VEGF) expression [80]. Moreover, in a murine model of 6-hydroxydopamine (6-OHDA)-induced sympathetic denervation, VIP administration effectively restored impaired fracture healing [40]. Collectively, these findings support a protective and pro-regenerative role for VIP in skeletal remodeling and repair. In contrast to the pro-regenerative effects of VIP, DA signaling exhibits conflicting results in rat BMSC *in vitro* models. One study reported that DA promoted osteogenic differentiation through dopamine receptor D1 (D1R)-mediated ERK activation [81]. By contrast, another investigation showed that DA suppressed osteogenesis by inhibiting the Akt/glycogen synthase kinase (GSK)-3β/β-catenin pathway [51]. This pronounced discrepancy may be associated with DA’s non-selective receptor binding and the engagement of distinct DR subtypes that trigger divergent downstream signaling cascades (Table 1) [14], [34], [40], [51], [73], [74], [75], [77], [78], [80], [81], [82], [83].

**Table 1:** Autonomic nerves and associated mediators exert diverse biological effects on MSCs through distinct signaling mechanisms.

**Autonomic neurotransmitter and neuropeptide signaling**	**Receptors**	**Signaling pathways**	**Experimental model systems**	**Biological functions**	**References**
SNS	β1-AR	MAPK-ERK1/2/JNK/p38	Normotensive (Wistar) and spontaneously hypertensive rats	Early osteogenic differentiation, expression of Runx2, osterix, and β-catenin↑	[14]
	β2-AR	NA	BMSCs of New Zealand rabbits	Osteogenic differentiation↓; Proliferation, migration↑	[75]
PSNS	nAChR	NA	Human BMSCs	Spontaneous migration↑	[78]
SNS/NE	β2-AR	MAPK-ERK1/2; cAMP-PKA	Human BMSCs	Proliferation in high NE density↓	[73]
	β3-AR	NA	Rats underwent TCST	SDF-1-induced migration↓; Osteogenic differentiation↓; Expression of ALP, OCN, and Runx2↓	[82]
	α-AR	Akt/Bcl-2	Diabetic mice underwent SYMX; Mouse BMSCs	Apoptosis induced by high glucose↓; ROS induced by high glucose↓	[74]
ACh	m1AChR	Ca^2+^-PKCζ-ERK1/2	Rat BMSCs	Migration induced by ACh in a time- and dose-dependent manner↑	[77]
NPY	Y1R	cAMP-PKA-CREB	Osteocyte-specific *NPY* knockout mice (Dmp1-iCre; NPY^f/f^); NPY^f/f^ control mice; Osteoblast-specific Cre mice (Col1a1-CreERT2; NPY^f/f^)	Adipogenic differentiation↑; Osteogenesis↓; Bone loss and marrow adiposity accumulation↑	[34]
	Y1R	NA	Rat BMSCs; Femoral fracture model in rats	Proliferation↑; Migration↑; Osteoblastic differentiation↑; Expression of ALP, COL1A1, OCN, and Runx2↑	[80]
SNS/VIP	NA	NA	Chemical SYMX mouse model; Mouse femur transverse fracture model	*In vitro* osteogenic differentiation↑; Expression of OCN and OPN↑	[40]
	VPAC1/2	Wnt-β-catenin-Runx2; cAMP signaling pathway	Rat BMSCs; Human BMSCs	Expression of OPN, OCN, and VEGF↑; Osteogenic differentiation↑	[83]
DA	NA	Akt-GSK-3β-β-catenin	Rat BMSCs	Osteogenic differentiation↓; Adipogenic differentiation↑	[51]
	D1R	cAMP-PKA-ERK1/2-Runx2	Human BMSCs	Low DA density (5 nmol/L): osteogenic differentiation↑; High DA density (≥5 nmol/L): osteogenic differentiation↓	[81]

↑ Upregulation; ↓ Downregulation. NA. Not applicable; SNS. Sympathetic nervous system; PSNS. Parasympathetic nervous system; NE. Norepinephrine; Ach. Acetylcholine; NPY. Neuropeptide Y; VIP. Vasoactive intestinal peptide; DA. Dopamine; AR. Adrenergic receptor; nAChR. Nicotinic acetylcholine receptor; m1AChR. Muscarinic acetylcholine receptor m1; Y1R. Neuropeptide Y receptor type 1; VPAC. Vasoactive intestinal peptide receptor; D1R. Dopamine receptor D1; MAPK. Mitogen-activated protein kinase; ERK. Extracellular signal-regulated kinase; JNK. c-Jun N-terminal kinase; p38. p38 mitogen-activated protein kinase; cAMP. Cyclic adenosine monophosphate; PKA. Protein kinase A; Akt. Protein kinase B; Bcl-2. B-cell lymphoma 2; PKCζ. Protein kinase C zeta; CREB. cAMP response element-binding protein; Wnt. Wingless-related integration site; Runx. Runt-related transcription factor; GSK. Glycogen synthase kinase; BMSCs. Bone marrow mesenchymal stem cells; TCST. Transcutaneous electrical spinal cord stimulation; SYMX. Sympathectomy; SDF. Stromal cell-derived factor; ALP. Alkaline phosphatase; OCN. Osteocalcin; ROS. Reactive oxygen species; VEGF. Vascular endothelial growth factor; COL1A1. Collagen type I alpha 1 chain

### 3.2 Osteoblast differentiation

Osteoblasts are chiefly derived from BMSCs, with some eventually differentiating into osteocytes embedded in the bone matrix. These cells are critical for regulating the bone microenvironment [84], [85]. In addition, osteoblasts largely promote the deposition of nascent extracellular matrix (ECM) through the secretion of extracellular proteins, such as type I collagen, osteocalcin (OCN), osteopontin (OPN), and alkaline phosphatase (ALP), thereby mediating bone formation [29], [86]. Current studies emphasize the critical involvement of the ANS in the development and regulation of the function of osteoblasts [86], [87], [88], [89]. For instance, in the bone microenvironment, adrenergic signaling from the SNS stimulates osteoblasts to secrete active factors such as interleukin (IL)-6, IL-1β, IL-11, tumor necrosis factor (TNF), prostaglandin E2 (PGE2), and vascular endothelial growth factor A (VEGF-A) [90], [91], [92]. However, cholinergic signaling from the PSNS may directly drive the proliferation and differentiation of osteoblasts [93], [94].

The molecular basis for this neuro-osteogenic communication lies in direct receptor engagement. Recent studies have confirmed that α-ARs, β-ARs, nAChRs, and mAChRs are expressed on the surface of osteoblasts [95], [96], [97], [98], [99]. Among these receptors, the β2-ARs act as the principal transducers of systemic sympathetic signals. Downstream of the receptors, molecular clock genes were identified in period circadian regulator 1 and period circadian regulator 2 double-knockout (Per1/2^−/−^) mouse models as essential mediators of leptin-driven suppression of bone formation. In osteoblasts, SNS-β2-AR signaling simultaneously activated two mechanistically antagonistic pathways. Specifically, one pathway constituted a predominant inhibitory axis, in which cAMP response element-binding protein (CREB)-dependent upregulation of clock genes suppressed the cellular myelocytomatosis oncogene (c-Myc) and cyclin D1, thereby arresting cell-cycle progression. Conversely, a parallel pathway mediated by activator protein 1 (AP-1) promoted proliferation [96], [100]. Mechanistically, the molecular clock functioned upstream of the AP-1 pathway, exerting a sustained repressive effect on AP-1 expression. Indeed, in *Per1/2*-deficient mice, the loss of *Per1/2* repression augmented AP-1-driven proliferation, resulting in a high-bone-mass phenotype. Consequently, under normal physiological conditions, clock-mediated suppression overrode anabolic signaling, leading to net bone loss within the leptin-sympathetic axis [96], [100]. Upstream of this clock mechanism, the loss of *β2-ARs* in Adrβ2^osb−/−^ mice similarly resulted in a high-bone-mass phenotype. Furthermore, although intracerebroventricular leptin infusion suppressed bone formation in wild-type mice, this anti-osteogenic effect was completely abolished in *Adrβ2*-knockout animals [96], [100]. Collectively, these findings confirm that central leptin-driven sympathetic signaling relies on osteoblastic β2-ARs to restrain bone accrual. Therefore, we propose that central leptin restrains bone formation via a sequential SNS-NE-β2-AR-clock gene axis in osteoblasts. This model can be directly tested by subjecting osteoblast-specific *β2-ARs* or *Per1/2* knockout mice to intracerebroventricular leptin infusion to determine whether disruption of this axis abolishes leptin-induced suppression of bone formation.

In contrast, increasing evidence highlights α-AR signaling as a potent stimulator of osteogenesis, contrasting with the canonical inhibitory role of β2-ARs. In human SaM-1 osteoblasts, NE binding to α1B-ARs triggered dual mitogenic cascades, a Gq-coupled phosphoinositide-specific phospholipase C (PI-PLC)/Ca^2+^ axis and a Gi/o-Gbɡ-mediated suppression of K^+^ channels, which synergistically promoted osteoblast proliferation [101]. These anabolic effects were further corroborated by *in vivo* murine models, demonstrating that α1B-AR activation upregulated the transcription factor CCAAT/enhancer-binding protein delta (EBPδ) to directly facilitate osteoblast expansion and bone accrual [97]. Furthermore, beyond these post-synaptic pro-osteogenic effects, pre-synaptic α2-ARs also critically regulate skeletal homeostasis. For instance, the dual disruption of α2A- and α2C-ARs in mice led to excessive NE release and a paradoxically high bone mass phenotype [95]. This anomaly is primarily driven by compensatory hyperinsulinemia, which ultimately overrides direct catecholaminergic inhibition. Taken together, these findings reveal that SNS-α-AR signaling exerts a profound anabolic influence on osteoblasts.

Moreover, the mAChRs (M1–M5) were widely expressed in human primary osteoblasts and bone tissue, where they drove osteoblast proliferation via amplifying intracellular calcium signaling [102]. Conversely, *in vitro* murine MC3T3-E1 models revealed paradoxical nicotinic effects. Specifically, exogenous nicotine stimulated cyclin D1-driven proliferation yet arrested p53-mediated osteogenic differentiation and mineralization. This process stalled osteoblasts at an immature stage, ultimately resulting in bone loss [102], [103]. These conflicting cholinergic effects highlight a critical limitation of current neuroskeletal models. Importantly, this arrest in differentiation likely reflects pathological nicotinic receptor overactivation, similar to smoking-induced bone loss, rather than the physiological role of endogenous ACh, which relies on the coordinated action of both receptor types.

In a stress model using *NPY/Y2R* knockout mice, sympathetic signaling via NPY-Y2R suppressed NE release, which reduced the stress-induced inhibition of osteoblast activity and partially prevented stress-related bone loss [104]. This work is the first to demonstrate the protective role of NPY in stress-induced bone loss, suggesting a potential strategy for treating stress-related OP, including depression-associated bone loss. Other studies have shown that VIP binds to VPAC2 receptors on osteoblasts, activating G protein-coupled signaling, increasing intracellular cyclic adenosine monophosphate (cAMP), triggering the PKA pathway, increasing ALP activity, promoting bone matrix mineralization, and ultimately supporting osteogenesis [44], [102], [105]. Furthermore, the activation of D1R on MC3T3-E1 osteoblasts *in vitro* increased ERK1/2 phosphorylation and reversed dexamethasone-induced suppression of osteogenesis, whereas *in vivo* activation of D1Rs alleviated glucocorticoid-induced bone loss in rats [106]. These findings suggest that D1R may be a potential target for glucocorticoid-induced OP, although interventions directly targeting DA as a neurotransmitter have not been investigated. Overall, the current evidence indicates that NPY, VIP, and DA can promote osteoblast proliferation and differentiation, suggesting potential neural-based strategies to increase bone formation and mitigate bone loss.

### 3.3 Osteoclast activity

Osteoclasts originate from HSC-derived myeloid mononuclear precursors and participate in bone remodeling primarily by driving bone resorption [107]. Recently, the regulatory role of the ANS in osteoclastogenesis has garnered significant attention [5], [108], [109], [110]. Within the bone microenvironment, osteoclast maturation and activation fundamentally depend on key local factors, such as receptor activator of nuclear factor kappa-B ligand (RANKL), osteoprotegerin (OPG), macrophage colony-stimulating factor (M-CSF), IL-6, and so on [111], [112], [113]. Crucially, rather than being synthesized directly by nerve terminals, the expression of these factors is profoundly modulated by sympathetic efferent signals. Multiple studies have demonstrated that osteoclast maturation is primarily driven by osteoblast- and chondrocyte-derived RANKL under the control of the SNS-NE-β2-AR signaling pathway [107], [114]. Specifically, the leptin-SNS-NE-β2-AR axis was shown to activate the PKA-activating transcription factor 4 (ATF4) pathway in osteoblasts, leading to increased RANKL expression, which subsequently facilitated intercellular cross-talk to promote osteoclast precursor differentiation [115], [116]. Supporting this indirect regulatory mechanism, low-dose propranolol (0.1 mg/kg) not only alleviated local inflammation by downregulating IL-1β and TNF-α but also decreased RANKL and elevated OPG levels, thereby downregulating osteoclast-related genes [tartrate-resistant acid phosphatase (*TRAP*), *cathepsin K*, and matrix metalloproteinase (*MMP*)-*9*] via the nuclear factor of activated T cells cytoplasmic 1 (NFATc1) pathway [108]. This finding highlights the dual anti-inflammatory and anti-osteoclastogenic efficacy of β-blockers in OP therapy.

Beyond indirect regulation via osteoblast-derived RANKL, accumulating evidence suggests that the SNS exerts direct effects on osteoclasts [109], [110]. *In vitro* studies have revealed that mature osteoclasts and their precursors express specific autonomic receptors, predominantly the β2-AR subtype [109], [110]. Direct stimulation with isoproterenol (ISO) increased intracellular ROS levels, subsequently upregulating NFATc1 and other osteoclast-related genes to promote osteoclastogenesis [110]. Furthermore, in stress models utilizing *β2-AR*- or *miR-21*-knockout mice, sympathetic stress directly increased osteoclastic miR-21 expression via β2-AR signaling, which inhibited programmed cell death protein 4 (Pdcd4) and resulted in pronounced bone loss [109]. Conversely, the activation of endogenously expressed α2-ARs on the osteoclast surface acted as a brake, suppressing RANKL-induced NFATc1, TRAP, and cathepsin K expression to curtail osteoclastogenesis [114].

Based on these findings, we hypothesize a concentration-dependent, dual-receptor gating model. Under conditions of chronic stress or inflammation characterized by elevated NE levels, low-affinity β2-ARs become the dominant functional receptors. Their activation drives bone resorption through a two-pronged mechanism that indirectly upregulates the osteoblastic PKA-ATF4-RANKL axis and directly enhances osteoclastic ROS/miR-21 signaling. In contrast, under physiological or low-NE conditions, catabolic β2-AR signaling remains relatively quiescent. During these basal states, high-affinity α2-ARs on osteoclasts act alongside presynaptic negative feedback to restrain excessive bone resorption.

In contrast to the primarily catabolic effects of the SNS, parasympathetic cholinergic signaling was demonstrated to rely on ACh to counteract bone resorption by directly inducing osteoclast apoptosis [99], [117]. This protective mechanism is mediated by specific cholinergic receptors expressed on the osteoclast surface. For instance, activation of nAChRs inhibited RANKL-induced calcium oscillations, triggering osteoclast apoptosis *in vivo* [117]. Similarly, muscarinic signaling was found to transduce parallel protective effects. Specifically, the M3 muscarinic acetylcholine receptors (M3Rs) acted as a pivotal downstream target for PSNS-derived ACh. Corroborating the functional importance of this ACh-M3R axis, *M3R*-deficient mice exhibited increased bone resorption parameters, including an enlarged osteoclast surface area [98]. These observations indicate that baseline cholinergic tone exerts a crucial restraining influence on osteoclastogenesis. Ultimately, the SNS and PSNS orchestrate osteoclast differentiation and activity through a delicate balance of antagonistic interactions to maintain dynamic skeletal homeostasis.

Moreover, another neuropeptide, VIP, can bind to pituitary adenylate cyclase-activating polypeptide type I receptor (PAC1) receptors to suppress osteoclast recruitment, a process associated with the upregulation of OPG and the downregulation of RANKL and RANK expression [118], [119]. Furthermore, studies in both animal and human cell models revealed that VIP not only inhibited osteoclast differentiation via the ERK and nuclear factor κB (NF-κB) signaling pathways, but also negatively modulated NFATc1, hence exerting anti-osteoclastogenic effects [45], [119]. Specifically, DA could inhibit the maturation and activation of osteoclasts by suppressing the RANKL signaling pathway and reducing their bone-resorptive activity [120], [121]. DA binding to dopamine receptor D2 (D2R) reduced intracellular cAMP levels and suppressed RANKL-induced cellular proto-oncogene fos (*c-Fos*) and *NFATc1* mRNA expression in human osteoclast precursors, thereby inhibiting osteoclastogenesis [122]. Additionally, DA was shown to inhibit osteoclast differentiation via the D2R/cAMP/PKA/CREB signaling pathway [16].

Unlike the NE-β2-AR axis, which promotes osteoclast formation and activity, sympathetic signals mediated by VIP and DA tend to be inhibitory, indicating that osteoclast function is finely tuned by multiple sympathetic inputs to maintain bone homeostasis (Table 2) [45], [75], [96], [98], [100], [106], [108], [109], [114], [115], [117], [123], [124], [125].

**Table 2:** Autonomic nerves and related mediators mediate complex cellular responses in osteoblasts and osteoclasts via specific signaling pathways.

**Autonomic neurotransmitter and neuropeptide signaling**	**Receptors**	**Signaling pathways**	**Experimental model systems**	**Biological functions**	**References**
Osteoblasts					
SNS	β2-AR	CREB-c-Myc	Adrb2^osb-/-^, Creb^osb-/-^, Atf4^osb-/-^, and c-Myc^osb-/-^ mice	Proliferation↓; Expression of cyclin D1/D2/E1, c-Myc, and Per1↓	[96]
SNS/NE	β2-AR	NA	Osteoblasts of New Zealand rabbits	Proliferation ↑; Migration↑	[75]
Leptin (CNS)/SNS/NE	β2-AR	CREB-Per1/2/Cry1/2-c-Myc-cyclin D1; AP-1-c-Myc-cyclin D1	*Per1/2*- and *Cry1/2*-deficient mice	Osteoblast proliferation in the 1st pathway↓; Osteoblast proliferation in the 2nd pathway↑	[100]
	α1-AR	Cebpd-Ccne1	α1B^-/-^ mice	Proliferation↑	[97]
PSNS	M_3_R	NA	*M*_*1*_*R*^-/-^, *M*_*2*_*R*^-/-^, *M*_*3*_*R*^-/-^, *M*_*4*_*R*^-/-^, *M*_*3*_*R*^fl/fl^, α1(I)*collagen*-Cre and *Adrb2*^+/-^ mice	Bone mass accumulation↑; Proliferation↑	[98]
SNS/NPY	Y1R	cAMP signaling pathway	Col2.3GFPemd or Col2.3CFPc-yan mice	Differentiation↓; Mineralization↓	[125]
DA	D1R	MAPK-ERK1/2	Mouse BMSCs and MC3T3-E1 cells, overexpression and silencing of D1R in MC3T3-E1 cells, D1R-inhibited mice	Osteogenic differentiation↑; Expression of ALP, Runx2, and OSX↑; Glucocorticoid-induced bone loss↓	[106]
Osteoclasts					
SNS	β2-AR	NA	Murine osteoblasts/osteoclasts; Mice with bone loss	Osteoclast formation↑	[109], [124]
	β2-AR	NFATc1 signaling pathway	Rats with administered propranolol; RAW264.7 osteoclast precursor cells	Osteoclastogenesis *in vitro*↓; Expression of TRAP, cathepsin K, and MMP-9↓;	[108]
	α2-AR	cAMP-NFATc1; eIF2α signaling pathway	RAW264.7 osteoclast precursor cells; Mouse primary bone marrow cells; Mouse mature osteoclasts	Osteoclastogenesis↓; Expression of TRAP, cathepsin K↓	[114]
Leptin (CNS)/SNS/NE	β2-AR	ATF4-RANKL; CART pathway	Adrb2^osb-/-^, Creb^osb-/-^, Atf4^osb-/-^, c-Myc^osb-/-^ mice, *Adrb2*-deficient mice, ob/ob mice	Differentiation in the 1st signaling pathway, bone resorption in the 1st signaling pathway↑; Bone resorption in the 2nd signaling pathway↓	[115]
PSNS/ACh	nAChRs	RANK-Ca^2+^-NFATc1/c-Fos	nAChR subunit^-/-^ mice; Bone marrow-derived macrophages	Osteoclastogenesis in high nAChR agonist density↓; Osteoclastogenesis in low nAChR agonist density↑	[117]
NPY	Y1R	Gi protein/cAMP/RANKL	Mouse osteoclast-like cells	Isoprenaline-induced osteoclastogenesis↓	[123]
VIP	VPAC1/2	NFATc1 signaling pathway	Human peripheral blood mononuclear cells	Differentiation, fusion, bone resorption↓; Expression of DCSTAMP, CTSK, MMP-9, TRAP, and ITGB3↓	[45]

↑ Upregulation; ↓ Downregulation. NA. Not applicable; SNS. Sympathetic nervous system; CNS. Central nervous system; NE. Norepinephrine; PSNS. Parasympathetic nervous system; NPY. Neuropeptide Y; DA. Dopamine; ACh. Acetylcholine; VIP. Vasoactive intestinal peptide; AR. Adrenergic receptor; mAChR. Muscarinic acetylcholine receptor; Y1R. Neuropeptide Y receptor type 1; D1R. Dopamine receptor D1; nAChRs. Nicotinic acetylcholine receptors; VPAC. Vasoactive intestinal peptide receptor; CREB. cAMP response element-binding protein; c-Myc. Cellular myelocytomatosis oncogene; AP-1. Activator protein 1; cAMP. Cyclic adenosine monophosphate; MAPK. Mitogen-activated protein kinase; ERK. Extracellular signal-regulated kinase; NFATc1. Nuclear factor of activated T cells cytoplasmic 1; RANK. Receptor activator of nuclear factor kappa-B; ATF4. Activating transcription factor 4; CART. Cocaine- and amphetamine-regulated transcript; Cry1. Cryptochrome 1; BMSCs. Bone marrow mesenchymal stem cells; MC3T3-E1. MC3T3-E1 cells; RAW. Ralph, Abelson, and Watson; ALP. Alkaline phosphatase; OSX. Osterix; TRAP. Tartrate-resistant acid phosphatase; MMP-9. Matrix metalloproteinase 9; DCSTAMP. Dendritic cell-specific transmembrane protein; CTSK. Cathepsin K; ITGB3. Integrin subunit beta 3; Cebpd. CCAAT/enhancer-binding protein δ; eIF2α. Eukaryotic translation initiation factor 2 alpha

### 3.4 Chondrocytes homeostasis and differentiation

Chondrocytes, which originate from MSCs, are indispensable for preserving the structure and function of cartilage [126], [127]. Under physiological conditions, mature chondrocytes maintain cartilage homeostasis by synthesizing and remodeling ECM components [128], [129].

Recent studies have demonstrated extensive SNS innervation across cartilage, subchondral bone, and synovial tissues [130], [131]. Consistent with this distribution, functional ARs are expressed on human articular chondrocytes, subchondral bone cells, and synovial fibroblasts [18], [131], [132], [133], [134]. Mechanistically, sympathetic signaling profoundly impairs chondrogenesis and joint homeostasis, a process well documented across multiple models. Providing the most direct clinical relevance, human MSCs and patient-derived chondrocyte progenitor cells exhibited sustained β2-AR expression during chondrogenesis. Following local NE-mediated β2-AR engagement, SRY-related HMG-box transcription factor 9 (SOX9) expression was downregulated, suppressing cartilage-specific matrix synthesis [type II collagen and sulfated glycosaminoglycans (sGAGs)] and upregulating hypertrophic markers (type X collagen and MMP-13), thereby impeding cartilage formation and driving hypertrophic differentiation [135]. Complementing these human cellular phenotypes, murine *in vivo* and primary cell models have further elucidated the underlying intracellular signaling cascades. In primary murine rib growth plate chondrocytes, β-AR agonist ISO stimulation activated PKA and ERK1/2 signaling, induced Jun-B expression, and suppressed SOX6 transcription, leading to decreased type II collagen levels [135]. However, this study reflects mainly developmental chondrogenesis and is less informative for adult cartilage degeneration or OA pathology. In naturally aged OA mouse models, the local NE-β1-AR axis disrupted subchondral bone homeostasis by suppressing Sirtuin 6 (Sirt6) and promoting miR-125 upregulation in chondrocytes [136]. Furthermore, the specific mechanisms underlying systemic sympathetic influence have been elucidated using established cell lines. *In vitro* experiments utilizing the ATDC5 cell line demonstrated that epinephrine binding to chondrocytic β2-ARs activated the cAMP/PKA pathway, resulting in reduced SOX9/5/6-dependent expression of matrix genes and impaired chondrogenesis [137]. Although epinephrine is not directly released from local sympathetic terminals, preganglionic sympathetic activation allows ACh to stimulate adrenal medullary chromaffin cells, thereby inducing systemic epinephrine secretion to indirectly govern chondrocyte function. Collectively, these converging findings establish the sympathetic-skeletal axis as a pivotal catabolic driver of cartilage degeneration. Operating through both local neural networks and systemic endocrine signals, aberrant adrenergic activation profoundly disrupts chondrocyte homeostasis.

In a destabilization of the medial meniscus-induced OA mouse model, NPY activated mechanistic target of rapamycin complex 1 (mTORC1) through neuropeptide Y receptor Y2 (NPY2R), which triggered Sma and Mad-related protein (SMAD)1/5/8 phosphorylation and subsequently aggravated cartilage degeneration and chondrocyte hypertrophy [31]. These observations indicate that the selective blockade of NPY2R may be a feasible therapeutic strategy for OA. Moreover, in terminally hypertrophic chondrocytes in mice, the binding of leptin to its receptor inhibited premature matrix mineralization, preserved the ordered arrangement of collagen fibrils, stabilized type X collagen expression, modulated terminal differentiation, and attenuated the excessive apoptosis of hypertrophic chondrocytes through multiple regulatory pathways [138], [139]. Additionally, leptin treatment increased insulin-like growth factor 1 receptor expression in growth plates and mandibular condylar cartilage, thereby facilitating skeletal growth [140]. Collectively, the current evidence suggests that NPY and leptin are involved in maintaining cartilage homeostasis by modulating chondrocyte proliferation, hypertrophy, and matrix metabolism.

### 3.5 Nucleus pulposus cell metabolism

NPCs originate from the embryonic notochord and mainly consist of notochordal- and chondrocyte-like populations, forming the principal cellular components of the nucleus pulposus [141]. In the mature disc, NPCs are embedded in a matrix-rich environment and display round, oval, or polygonal morphologies. Due to their resemblance to chondrocytes, they are often termed chondrocyte-like cells [20]. Functionally, NPCs maintain ECM homeostasis and contribute to the immune and internal microenvironment of the intervertebral disc [142], [143], [144]. Disruption of their metabolic activity or cellular function contributes to the initiation and progression of IVDD [19].

NPCs are modulated by sympathetic neurotransmitters and their receptors, including NE and NPY [145], [146]. Furthermore, in cultured human intervertebral disc cells, transcripts for almost all AR subtypes were detected, except for α1D-AR and β3-AR [147]. NE was reported to promote matrix catabolism in annulus fibrosus cells *in vitro* through activation of the ERK1/2 and PKA pathways, although the specific AR subtypes involved remain undefined [147]. In addition to NE, NPY affects NPC biology, but its precise molecular mechanisms were not fully elucidated. Further evidence indicated that NPY suppressed mitochondria-dependent apoptotic signaling, promoted NPC proliferation, protected cells from IL-1β-induced apoptosis, and decreased the expression of catabolic proteases, thereby potentially attenuating IL-1β-induced matrix degradation [20]. These observations suggest that targeting NPY may delay the progression of IVDD by preserving NPC viability. Taken together, the current evidence supports the hypothesis that ANS signaling is actively involved in modulating NPC behavior during disc degeneration.

### 3.6 Hematopoietic stem cells, adipocytes, and tendon cells

HSCs reside in specialized bone marrow niches, maintaining their capacity for self-renewal and multilineage differentiation. Recent studies have identified bone marrow hematopoietic stem cells (BMHSCs) as key contributors to fracture healing [79], [148], [149]. Within this microenvironment, their migration, self-renewal, and differentiation are tightly governed by sympathetic innervation. Specifically, β3-AR-mediated sympathetic signaling increased HSC proliferation and promoted their differentiation into emergency myeloid cells, thereby supporting fracture repair [148]. Conversely, the disruption of this adrenergic pathway not only reduced sympathetic nerve-driven inflammatory myeloid skewing [150], [151] but also accelerated HSC aging, compromising overall hematopoietic function [152]. Alongside these adrenergic mechanisms, chronic stress elevated NPY levels within the bone marrow. Upon binding to osteoblastic Y1Rs, NPY modulated MMP-9 secretion, reducing the expression of hematopoietic stem and progenitor cell (HSPC) maintenance factors such as stromal cell-derived factor (SDF)-1α, Kit ligand (Kitl), Angiopoietin 1 (Angpt1), and Vascular cell adhesion molecule 1 (Vcam1), and promoting HSPC mobilization into the peripheral circulation. Consequently, this mobilization decreased osteoclast numbers and alleviated ovariectomy-induced bone loss in mice [153]. However, as this study did not assess whether NPY directly alters intrinsic HSPC properties, such as cell cycle progression or cellular senescence, the contribution of HSPC-autonomous regulation to this mobilization process cannot be entirely ruled out.

Bone marrow adipose tissue (BMAT) within the skeleton and marrow cavity is a major cellular component of the bone marrow niche and skeletal microenvironment [113], [154]. Previous studies have indicated that a subset of BMAT is modulated by the ANS, and that bone marrow adipocytes express large amounts of β-ARs [155], [156]. Upon NE stimulation, these receptors activate the cAMP-PKA pathway, trigger lipolysis, and promote the release of free fatty acids and glycerol, thereby supporting the metabolic demands of cells in the bone marrow microenvironment [157], [158], [159]. Moreover, VIP was reported to induce lipolysis in primary rat and human adipose tissues *in vitro* [160], [161], [162].

**Fig. 3:**
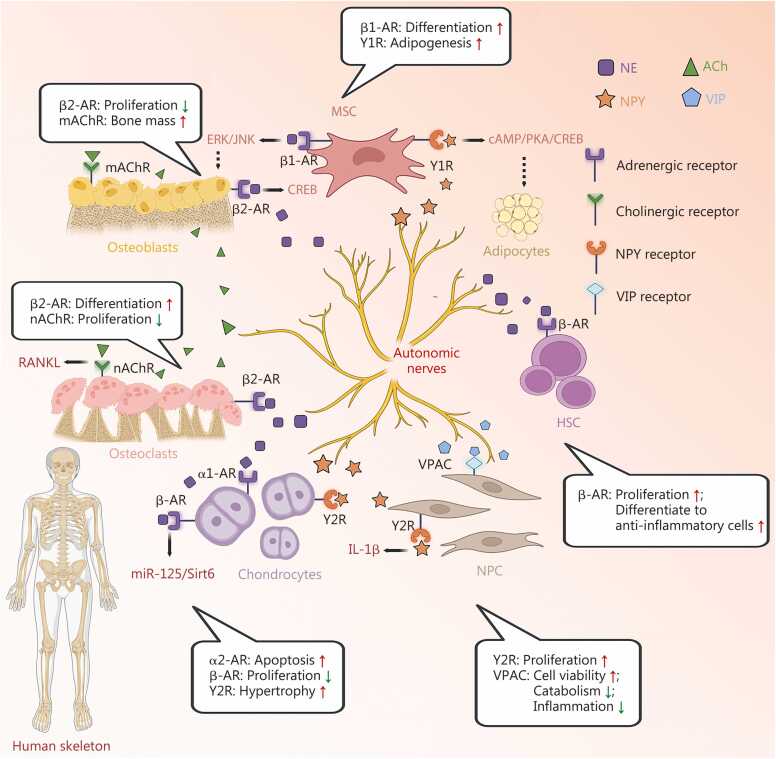
Autonomic nerves and their neurotransmitters/neuropeptides regulate the proliferation, differentiation, and formation, etc., of mesenchymal stem cells (MSCs), osteoblasts, osteoclasts, chondrocytes, nucleus pulposus cells (NPCs), hematopoietic stem cells (HSCs), and adipocytes in bone metabolism. Created with BioRender.com. ↑ Upregulation; ↓ Downregulation. VIP. Vasoactive intestinal peptide; NPY. Neuropeptide Y; ACh. Acetylcholine; NE. Norepinephrine; ERK. Extracellular signal-regulated kinase; JNK. c-Jun N-terminal kinase; cAMP. Cyclic adenosine monophosphate; PKA. Protein kinase A; CREB. cAMP response element-binding protein; AR. Adrenergic receptor; mAChR. Muscarinic acetylcholine receptor; nAChR. Nicotinic acetylcholine receptor; Y1R. Neuropeptide Y receptor type 1; VPAC. Vasoactive intestinal peptide receptor; IL. Interleukin.

In addition to BMAT, tendon tissue also receives ANS innervation. During tendon injury and repair, multiple types of nerve fibers, including autonomic fibers, extend into the tendon proper and contribute to tissue regeneration [163], [164], [165]. The SNS regulates tendon function partly through the modulation of local blood flow, whereas the PSNS influences inflammatory responses in tendon tissue [164]. Furthermore, ACh was shown to increase tendon cell proliferation [166]. In summary, previous studies on ANS regulation of BMAT and tendon tissue have been limited and largely focused on the local effects of individual neurotransmitters or receptor subtypes. At present, a systematic understanding of the regulatory mechanisms in these two tissues is lacking. Moreover, the coordinated neural circuits involved in maintaining tissue homeostasis and supporting repair and regeneration remain insufficiently defined and warrant further investigation (Fig. 3, Table 3) [18], [20], [31], [131], [135], [136], [147], [148], [153], [167].

**Table 3:** Autonomic nerves and related mediators orchestrate complex biological effects in chondrocytes, NPCs, and HSCs through distinct signaling mechanisms.

**Autonomic neurotransmitter and neuropeptide signaling**	**Receptors**	**Signaling pathways**	**Experimental model systems**	**Biological functions**	**References**
Chondrocytes					
SNS/NE	β2-AR	NA	Peripheral sympathectomy, OA-induced mice	OA-specific cartilage calcification↑	[131]
	β2-AR	AP-1 (Jun-B)	Growth plate chondrocytes prepared from ribs of embryonic E18.5 mice	Expression of type II collagen↓; Cartilage matrix synthesis↓	[135]
	α1-AR and β-AR	NA	Human OA cartilage chondrocytes	Expression of IL-8, MMP-13, GAG, type II collagen in high NE density (10^-^⁶ mol/L), proliferation in in high NE density↓; Proliferation and apoptosis in low NE density (10^-8^ mol/L)↑	[18]
	β1-AR	Sirt6/miR-125	Mouse natural aging model, a medial, and a load-induced meniscus instability model	Cartilage matrix degradation, cartilage damage↑	[136]
SNS/NPY	Y2R	mTORC1-SMAD1/5/8-Runx2	Mouse OA model	Chondrocyte hypertrophy, matrix degradation↑; Chondrocyte autophagy↓; Chondrocyte apoptosis↑	[31]
NPCs					
SNS/NE	α-AR and β-AR	ERK1/2 signaling pathway; PKA signaling pathway	Human IVD tissue and murine IVDD models	Unclear	[147]
NPY	Y2R	IL-1β pathway	Rat NPCs	Proliferation↑; IL-1β-induced cell apoptosis, IL-1β-induced matrix degradation↓	[20]
VIP	NA	NA	Human NPCs	Cell viability, expression of aggrecan and col2a1↑; Expression of pro-apoptotic (*Bax*) and catabolic genes (*MMP-3/13*) and inflammation-related genes↓	[167]
HSCs					
SNS	β2-AR and β3-AR	NA	Adrb2^-/-^, Adrb3^-/-^ mice	Proliferation↑; Skews HSCs toward anti-inflammatory myeloid cells↑	[148]
NPY	Y1R	NA	Lyz2-cre, 2.3-kB col1a1-cre, and Y1^fl/fl^ mice	Hematopoietic stem/progenitor cell mobilization↑	[153]

↑ Upregulation; ↓ Downregulation. NA. Not applicable; SNS. Sympathetic nervous system; NE. Norepinephrine; AR. Adrenergic receptor; OA. Osteoarthritis; AP-1. Activator protein 1; Jun-B. Jun B proto-oncogene; IL. Interleukin; MMP. Matrix metalloproteinase; GAG. Glycosaminoglycan; Sirt6. Sirtuin 6; miR-125. MicroRNA-125; Y2R. Neuropeptide Y receptor type 2; NPY. Neuropeptide Y; mTORC1. Mechanistic target of rapamycin complex 1; SMAD. Sma and Mad related protein; Runx. Runt-related transcription factor; ERK. Extracellular signal-regulated kinase; PKA. Protein kinase A; IVD. Intervertebral disc; IVDD. Intervertebral disc degeneration; VIP. Vasoactive intestinal peptide; Bax. Bcl-2-associated X protein; NPCs. Nucleus pulposus cells; HSCs. Hematopoietic stem cells

## 4 The role of the autonomic nervous system and clinical implications in bone diseases

### 4.1 Osteoporosis

OP is a systemic skeletal disease characterized by a decrease in bone density, which leads to an increased susceptibility to fractures from minor falls, particularly in weight-bearing areas such as the spine and hip [168]. This condition predominantly affects the elderly and postmenopausal women. The resulting economic burden and consumption of social resources have made OP a major global public health concern [105]. Epidemiological data indicate that approximately 200 million individuals worldwide are affected by this disease [169]. The onset and progression of OP are regulated by osteoclast-mediated bone resorption and osteoblast-mediated bone formation [170]. Its treatment involves mainly pharmacological interventions, including antiresorptive medications (such as bisphosphonates and denosumab) and bone-forming agents (such as teriparatide) [171]. Although these treatments are effective, they are associated with certain adverse effects such as thromboembolic events, an increased stroke risk, and hypocalcemia, highlighting the urgent need for the development of safer and more effective therapeutic strategies [172].

The ANS plays a key role in regulating skeletal growth and bone metabolism, particularly in OP development [173]. Numerous studies have demonstrated that the SNS and its primary neurotransmitter, NE, regulate bone homeostasis through β-ARs [96], [97], [109]. Typically, this regulatory mechanism promotes osteoclast formation and inhibits osteoblast function, resulting in increased bone resorption and decreased bone mass [174]. Furthermore, animal studies revealed that NE signaling suppressed parathyroid hormone (PTH)-induced osteoblast differentiation and mineralization, while promoting RANKL expression and osteoclast differentiation [88], [131]. However, the β-blocker propranolol can partially counteract these effects and enhance PTH-induced osteogenesis [88]. In contrast, the NE-β2-AR signaling pathway has been shown to promote osteoblast proliferation and bone formation by upregulating AP-1 and cyclin D1 expression *in vitro* [100], which contradicts the widely accepted view that the SNS inhibits osteogenesis. These apparently contradictory findings likely reflect context-dependent differences in β2-AR signaling. Under *in vitro* or acute stimulation conditions, transient β2-AR activation promotes osteoblast proliferation via AP-1 and cyclin D1. In contrast, under chronic stress or inflammatory conditions *in vivo*, persistently elevated NE levels enhance RANKL expression and osteoclastogenesis, thereby driving bone resorption. This hypothesis can be tested using *in vivo* models comparing acute low-dose and chronic high-dose NE stimulation in combination with β2-AR blockade to determine whether β2-AR signaling undergoes a time- and intensity-dependent functional shift.

The PSNS and its neurotransmitter ACh exert a protective effect on bone homeostasis. Epidemiological studies have shown that nicotine affects bone metabolism via ACh receptors, with low doses stimulating osteogenesis and high doses inhibiting bone remodeling, which may explain the association between smoking, decreased bone mass, and an increased fracture risk [175], [176]. Moreover, ACh regulates bone remodeling through both nAChR and mAChR, helping to maintain peak bone mass in adult mice [99], [103], [177].

Additionally, in an ovariectomized rat model, the blockage of Y1Rs significantly improved the bone microstructure, upregulated the osteogenic markers runt-related transcription factor 2 (Runx2) and OPG, and simultaneously reduced RANKL and MMP-9 levels [15]. Furthermore, the ECS also plays a role in bone metabolism, with activation of CB1 receptors tending to promote osteoclast activity and inhibit osteogenesis, whereas CB2 receptor activation tends to protect bone structure and maintain bone homeostasis [178]. Within this neuroendocrine framework, leptin, which is secreted by adipocytes, regulates food intake and energy metabolism through leptin receptors in the central hypothalamus [60], [179], [180], [181] and influences bone formation and resorption via neural pathways [182]. Mechanistically, leptin increases sympathetic activity in glucose-sensitive neurons in the ventromedial hypothalamus, which increases NE release and downregulates the expression of the osteoblast proto-oncogene c-Myc. This process results in cell cycle arrest at the G1 phase, inhibiting osteoblast proliferation [59], [100]. In addition, leptin-regulated sympathetic signaling promotes bone resorption by inducing RANKL expression in osteoblast progenitors via Adrb2 and ATF4 phosphorylation. Consistently, β2-receptor knockout mice exhibit high bone mass [115], supporting a role for leptin-SNS signaling in bone loss. In contrast, several studies have demonstrated direct peripheral effects of leptin on bone cells. For example, leptin inhibits osteoclast generation in cultures of human peripheral blood mononuclear cells, possibly through interaction with soluble RANKL and increased OPG expression [61]. Moreover, leptin synergizes with 25(OH)D₃ to promote osteogenic differentiation of rat MSCs [183]. These findings suggest that leptin may act locally to enhance bone formation and increase bone mass, thereby potentially reducing the risk of OP. Clinical studies have further examined the association between leptin and bone metabolism. In a study involving 121 healthy postmenopausal women, baseline leptin levels were positively correlated with spinal and hip bone mineral density (BMD) and were associated with reduced bone resorption [C-terminal telopeptide of type I collagen (CTX) levels decreased by 38%] and decreased bone formation markers [osteocalcin (OC) levels decreased by 21%] [184]. Even after adjusting for body mass index (BMI) or fat mass, the negative correlation between leptin and CTX levels persisted, indicating that leptin may be an independent factor involved in the peripheral suppression of bone resorption. In another cohort study of women, leptin levels were positively correlated with PTH levels, negatively correlated with 25-OH vitamin D levels, and strongly correlated with BMI [185]. More importantly, the classic “secondary hyperparathyroidism” phenomenon, in which a low vitamin D level is associated with high PTH levels, was observed only in the high leptin group. These interactions suggest that leptin may simultaneously suppress bone resorption and modulate the PTH response, thereby influencing bone metabolism under conditions of vitamin D deficiency. Therefore, targeting the inhibition of leptin secretion or production may serve as a potential therapeutic strategy. It could also aid in risk assessment and drug management for postmenopausal women with OP, providing a more accurate way to identify high-risk women and guide early intervention.

Emerging human clinical studies also provide preliminary evidence for other ANS-related interventions that aim to modulate bone metabolism. A case series involving postmenopausal women with bone loss revealed that oral cannabidiol (100–300 mg/d for 12 weeks) was well-tolerated and associated with reductions in bone resorption and formation markers, suggesting potential bone-protective effects [186]. More direct evidence was obtained from human studies on β-blockers. In a cohort study of 248 subjects and a randomized trial involving 155 postmenopausal women, β1-selective blockers (atenolol and nebivolol) improved the bone microarchitecture, reduced serum CTX levels, and increased the distal radius BMD, whereas the nonselective β-blocker propranolol showed no comparable effects [187]. These findings suggest that bone-protective effects depend on receptor selectivity and may reflect species-specific differences in adrenergic regulation. Another clinical study involving 944 women revealed that the use of β-blockers was associated with higher femoral neck and lumbar spine BMD, a lower risk of fractures, and a better trabecular structure [188]. Consistently, a nationwide case-control study in Denmark investigated the relationship between β-blocker use and fracture risk, and revealed that these drugs significantly reduced the fracture risk, particularly for hip fractures [189], supporting the efficacy of SNS inhibition in reducing bone resorption and the fracture risk.

In early postmenopausal women, the Leu7/Pro7 polymorphism of the NPY signaling gene was positively correlated with the femoral neck BMD. Five-year follow-up revealed that Leu7/Pro7 carriers in the non-hormone replacement therapy (non-HRT) group had less BMD loss, whereas HRT intervention did not significantly affect changes in the BMD of patients with this genotype [190]. In addition, patients with pheochromocytoma (a condition causing excessive catecholamine secretion and SNS overstimulation) had a 2.9% decrease in the lumbar spine trabecular bone score, reduced bone mass, and elevated levels of bone resorption markers. Urinary NE levels were negatively correlated with the lumbar spine bone microstructure and exhibited a dose-response relationship [191]. NPY may promote bone formation by regulating osteoblast activity and maintaining bone microstructure, whereas excessive SNS activation (elevated NE levels) accelerates osteoclast-mediated bone resorption and damages bone microstructure. Together with the above clinical data, interventions targeting SNS activity or NPY signaling may not only modulate the local balance between bone formation and resorption but also optimize bone homeostasis within the context of systemic neuro-endocrine regulation. Furthermore, OP patients may simultaneously experience ANS dysfunction. A prospective controlled trial revealed that OP patients exhibited increased sympathetic activity and decreased parasympathetic activity, which could further affect cardiac function [192].

Overall, OP involves not only abnormal bone metabolism but also systemic autonomic dysfunction, so interventions should aim to coordinate sympathetic and parasympathetic functions and their effects on other organs, rather than merely suppressing sympathetic activity.

### 4.2 Osteoarthritis

OA, which is characterized by cellular stress and ECM degradation, is one of the most prevalent joint disorders worldwide [193]. As of 2020, osteoarthritis affected approximately 595 million individuals worldwide, representing 7.6% of the global population, and cases are projected to approach or exceed one billion by 2050 due to population growth and ageing [194]. Unfortunately, an effective strategy to prevent or reverse OA is still unavailable. The standard treatment involves a combination of pharmacological and nonpharmacological therapies to alleviate symptoms, with joint replacement reserved as the last resort [195], [196], [197]. Although these treatments partially relieve symptoms and improve function, the effectiveness of pharmacological therapies remains limited, and these treatments can lead to adverse effects such as cardiovascular disease, gastrointestinal bleeding, and renal impairment [198], [199]. Furthermore, joint replacement carries risks, including catastrophizing, unrealistic expectations, and pain sensitization [198].

Recently, the potential role of the ANS in pathological changes associated with OA through neurotransmitter-receptor interactions has attracted increasing attention. Many studies have shown that the SNS is a crucial mediator of pathological changes in multiple joint tissues during the progression of OA [10], [131], [134], [200]. In a temporomandibular joint (TMJ)-OA rat model, abnormal occlusion activated the SNS, which further induced osteoclast activation and accelerated cartilage and bone degeneration [201]. Interestingly, propranolol (a β-AR antagonist) effectively inhibited this effect. Additionally, the PSNS is believed to play an important role in cartilage degeneration. Notably, nicotine activates the α7-nicotinic acetylcholine receptor (α7-nAChRs) on chondrocytes, effectively preventing cartilage degeneration and reducing proteoglycan loss [202], [203]. These findings highlight the potential therapeutic benefits of targeting specific receptor pathways in the regulation of cartilage degeneration. However, considering the potential damage to other organs caused by nicotine and similar agents, identifying an effective, nontoxic dose or alternative receptor agonist with greater safety and specificity would be beneficial.

Subchondral bone remodeling abnormalities are considered key factors involved in the progression of OA [204]. Specifically, subchondral bone sclerosis affects its ability to absorb and distribute mechanical loads, thereby accelerating cartilage degeneration [205]. Notably, increased SNS activity has been observed during OA progression, influencing both subchondral bone remodeling and cartilage degeneration [206]. NE-mediated SNS signaling can accelerate osteoblast senescence and disrupt subchondral bone homeostasis in aging mice [136]. In a mouse model of knee OA, inhibition of sympathectomy (SYMX) or knockout of *β2-AR* (Adrb2^-/-^) resulted in increased subchondral BMD, possibly because β2-AR activation inhibited osteoblast function while promoting osteoclast activation [131], [207]. Evidence across different OA models consistently indicates that SNS/β2-AR signaling suppresses osteoblast activity and promotes osteoclast activation, leading to subchondral bone loss, with differences among studies mainly reflecting variations in experimental models and intervention strategies.

Other sympathetic neuropeptides, such as VIP, have been shown to exert strong anti-inflammatory and immunomodulatory effects on cartilage degeneration and synovial inflammation [208], [209]. In a rheumatoid arthritis (RA) mouse model, VIP treatment significantly alleviated arthritic symptoms. Clinical observations revealed that the joint swelling and inflammation scores were markedly lower in the VIP treatment group than in the control group. Specifically, VIP treatment improved pathological changes in joints by downregulating the levels of proinflammatory cytokines such as TNF-α, IL-1, and IL-6, and inhibiting T helper (Th)1 cell responses [e.g., IL-12/interferon-γ (IFN-γ) production] [210], [211]. Additionally, VIP promoted the differentiation of Th2 cells, enhancing their anti-inflammatory effects [210], [211]. These findings provide important insights into the potential immunomodulatory effects of VIP, particularly for applications in rheumatoid diseases.

Recent studies have emphasized the role of NE in OA pathogenesis, showing that NE can regulate the metabolism and inflammatory response of human chondrocytes [18], [183]. Specifically, at a high concentration (10⁻⁶ mol/L), NE activates β-AR, reverses the IL-1β-induced increase in IL-8 and MMP-13, and restores the synthesis of type II collagen and GAGs, indicating potential anti-inflammatory effects. However, at a lower concentration (10⁻^8^ mol/L), NE induces apoptosis via the α1-AR signaling pathway [18]. Notably, the effects of NE are not limited to chondrocytes. In human MSCs and chondroprogenitor cells, NE-mediated β2-AR signaling may inhibit cartilage regeneration by suppressing the deposition of sGAGs and type II collagen [73], [212], [213]. These findings suggest that sympathetic NE signaling through β-AR may transiently suppress inflammation, yet persistent or imbalanced activation in degenerative cartilage shifts signaling toward apoptosis or impaired matrix synthesis. In contrast, vagus nerve stimulation (VNS)-mediated vagal activation primarily modulates systemic and central inflammation, potentially reducing pain and inflammatory burden without directly correcting cartilage degeneration. Furthermore, selective serotonin and NE reuptake inhibitors (SNRIs), such as milnacipran and duloxetine, exert significant analgesic effects during OA treatment. In a clinical study of the use of milnacipran in RA patients, the pain intensity was significantly reduced by 1.14 points after 6 weeks in a subgroup with a joint swelling count ≤1, compared with that in the placebo group [214]. This result supports the potential of sympathetic neurotransmitter accumulation in chronic pain management.

In addition, VNS, a direct method of modulating the ANS, has been shown to exert anti-inflammatory and analgesic effects on patients with RA. VNS modulates the vagus nerve to reduce peripheral and central inflammatory responses, representing a new therapeutic approach for OA. Both invasive and transcutaneous VNS stimulation significantly alleviate OA symptoms, including pain and inflammation [215]. Transcutaneous auricular VNS (tVNS) has therefore emerged as a potential therapeutic strategy for knee OA [216], possibly by correcting the reduced parasympathetic activity associated with the disease, although its precise mechanisms and effects on tissue degeneration require further investigation.

In summary, the ANS plays a crucial role in the complex processes of OA development and progression.

### 4.3 Fracture healing

A bone fracture refers to the disruption of bone integrity and continuity, typically caused by trauma or skeletal disease, and is often accompanied by localized pain, swelling, and functional impairment [217], [218]. With the steady increase in human life expectancy, age-related and osteoporotic fractures have become increasingly common and have emerged as major contributors to chronic pain and long-term disability worldwide [219], [220], [221]. These injuries not only impair physical function but also negatively impact mental health [222]. Therefore, identifying and optimizing strategies to promote fracture healing has become a pressing issue [223].

Recently, the potential role of the SNS in fracture healing has garnered increasing attention [40], [224]. In mice, the SNS forms a complex network widely distributed across cortical bone, trabecular bone, bone marrow, and periosteum, with the highest nerve density observed in areas of active bone formation, suggesting that the SNS may play a critical role in the early stages of fracture healing [25]. However, in sympathetic denervation experiments, mice with fractures exhibited a significant reduction in the trabecular bone volume and a marked decrease in bone mechanical strength, both in fractured and nonfractured bones [40], [225], [226]. This phenomenon highlights the importance of the SNS in bone homeostasis, particularly in the late stages of fracture healing, where the absence of sympathetic innervation not only delays callus maturation [227] but also significantly impairs mechanical stability [228].

Moreover, NE primarily promotes fracture healing through interactions with its receptors. Research has demonstrated that in a mouse model of femoral fractures and traumatic brain injury (TBI), the excessive sympathetic state induced by TBI enhances fracture repair via the NE-β2-AR signaling pathway [229], [230], [231]. These findings suggest that TBI may accelerate fracture healing by activating the SNS, providing new clinical insights for treating traumatic fractures. However, the effect of the SNS on fracture healing is not entirely beneficial. A study using a rat model of mandibular distraction osteogenesis with transection of the cervical sympathetic trunk has also shown that the NE-β3-AR signaling pathway negatively affects fracture healing under certain conditions, possibly by downregulating the expression of osteogenic genes such as *ALP*, *Runx2*, and *OCN* in MSCs, thereby inhibiting fracture repair [82]. Taken together, the effects of SNS signaling in fracture healing are determined by receptor subtypes and activation patterns. These results from animal models highlight the need for higher-quality human clinical trials to evaluate the therapeutic potential of ANS modulation in fracture healing.

A study using a chronic post-fracture pain mouse model has shown that enhancing parasympathetic signaling through nicotine administration, which augments the cholinergic system, effectively alleviates postoperative pain [232]. This suggests that manipulating ANS may play a crucial role in managing pain following traumatic fractures. Additionally, in a mouse femoral closed fracture model, significant amounts of sensory and sympathetic nerve fiber sprouting were observed at the nonhealed fracture site. The nerve fiber density increased, and structures resembling neuromas formed. This abnormal nerve growth not only poses a potential risk factor for fracture healing but also may contribute to ectopic skeletal pain [221]. In line with these findings, a randomized, double-blind study demonstrated that preoperative sympathetic block significantly reduced pain in patients undergoing upper limb orthopedic surgery, further emphasizing the importance of autonomic modulation in pain management [233].

The role of the SNS in bone repair has become a focal point of research. For instance, research has shown that wild-type mice subjected to fracture exhibit trauma-specific hyperinsulinemic stress responses and a decrease in OCN levels, which accelerates fracture healing. Leptin-deficient mice, however, lose the specificity of OCN changes, resulting in impaired fracture healing [234]. This observation aligns with previous research that underscores the promotive effects of the SNS on fracture healing [231]. Additionally, TBI is significantly associated with accelerated fracture healing [235]. Research by Wei *et al*. [236] revealed a positive correlation between serum leptin levels and callus volume at fracture sites in TBI patients. The marked increase in leptin concentration between weeks 4 and 12 post-trauma suggests that leptin may facilitate bone mineralization and osteoblast differentiation into osteocytes through peripheral effects, thereby accelerating fracture healing.

In addition to NE, NPY has garnered increasing attention for its role in bone repair, with several studies confirming its functional involvement [80], [237], [238]. Under physiological conditions, NPY-positive fibers are predominantly localized in blood vessels, with a sparse distribution in the periosteum [239], [240]. However, following a fracture, the number of NPY-positive fibers at the injury site significantly increases [237]. These findings suggest that NPY may play a key regulatory role in fracture healing. Animals with *Y1R* knockdown exhibit delayed early fracture healing, characterized by smaller callus volume, reduced mechanical strength, and insufficient vascularization [238], [241]. Conversely, *Y2R* knockout can increase the number of mesenchymal precursor cells in mice, increase osteogenic potential, and ultimately lead to an increased bone mass [33], [242]. The discrepancy in these findings may be attributed to differences in experimental design or the diversity of NPY receptor subtypes. Therefore, further research is needed to clarify the specific mechanisms of NPY in fracture healing. In mice with sympathetic nerve depletion induced by 6-OHDA, the femoral bone density, microstructure, and mechanical strength are significantly decreased, and fracture healing is markedly suppressed. However, local treatment with VIP led to significant improvements in bone microstructure and mechanical performance, with restored expression of osteogenic markers, such as OCN and OPN [40].

Overall, the SNS appears to exert a predominantly beneficial effect on traumatic fracture healing. Moderate sympathetic activation, particularly via β2-adrenergic signaling, promotes osteogenic differentiation and callus formation. In addition, leptin-related pathways are associated with increased callus volume and accelerated mineralization. Despite these promising findings, limitations still exist. Most studies have been conducted in animal models, for which no specific drugs targeting fractures through ANS modulation are yet available. Additionally, the optimal timing for treatment remains uncertain. For instance, the efficacy of reboxetine and VIP manifests primarily in the later stages of fracture healing, but the mechanisms underlying their early effects remain unclear.

### 4.4 Intervertebral disc degeneration

IVDD is defined as a progressive condition characterized by structural and functional deterioration of the disc, which is driven mainly by aging and repeated mechanical stress [243], [244]. IVDD has become a major global public health concern. In advanced stages, it often results in a wide range of disabling symptoms, such as chronic low back pain [245], [246], muscle weakness, reduced mobility, bowel or bladder dysfunction, and sensory disturbances in the extremities [247]. Globally, low back pain affected approximately 619 million people in 2020 and remained the leading cause of years lived with disability, with the number of prevalent cases projected to increase to 843 million by 2050 [194]. However, current therapies focus primarily on symptom relief and are largely ineffective at halting or reversing the degenerative process. Therefore, elucidating the underlying mechanisms of IVDD is crucial. Previous studies identified sympathetic nerve fibers and ARs in both healthy and degenerated disc tissues [147], [248], suggesting that the SNS might be involved in the pathogenesis and progression of IVDD.

In healthy intervertebral discs, sympathetic nerve fibers are located primarily around the annulus fibrosus, often in proximity to blood vessels [249], [250]. A study of rat cervical intervertebral discs has shown that sympathetic nerve fibers comprise approximately 20.4% of the total nerve fibers [251]. These findings suggest that SNS plays a role in the physiological function of the disc, possibly by regulating blood flow and cellular activity to help maintain normal disc metabolism. However, as the disc degenerates, a significant increase in the number of sympathetic nerve fibers occurs, and these fibers invade regions that were previously devoid of innervation, such as the inner annulus and even the nucleus pulposus [167], [251]. This change is not only pathological but also a hallmark of degenerative disc changes, suggesting that the SNS may accelerate the degenerative process. Increased AR expression appeared to be involved in pathological processes during IVDD. On the one hand, it is associated with catabolic activity and pain signaling mediated by neurotransmitters such as NE [252]. On the other hand, AR activation might exacerbate disc degeneration by enhancing hypertrophic changes in NPCs [167].

NPY and VIP are also increasingly recognized for their roles in IVDD. It has been demonstrated that the accumulation of NPY is significantly increased in degenerated rat intervertebral discs compared with naturally aging discs [19]. These observations suggest that the excessive accumulation of NPY may serve as a hallmark of disc degeneration. Dysregulation of the neuropeptide Y-Y receptor (NPY‑YR) axis could be a major driving factor for degenerative changes in the disc, particularly in processes involving inflammatory pain, disc cell apoptosis, and ECM degradation [253], [254]. Targeting this axis may significantly slow the degenerative process of the disc and alleviate the associated clinical symptoms, although clinical trials have not yet been conducted on neuroregulation-based treatments for IVDD.

Staining of human and mouse intervertebral disc tissues has shown that nearly all types of ARs are present in intervertebral discs at various stages of degeneration [147]. These findings suggest that the SNS plays an important role in the pathogenesis of IVDD, with different AR subtypes potentially playing distinct roles in the degenerative process. Brenneis *et al*. [252] further analyzed the expression of ARs in degenerative human intervertebral discs and correlated these findings with clinical parameters such as the Pfirrmann grade. The results showed that the expression of α1A-AR, α2A-AR, and β2-AR was significantly correlated with the Pfirrmann grade, suggesting the potential of ARs as prognostic markers for IVDD. Specifically, changes in the expression of these receptors reflect the severity of the degenerative process. However, the limitation of this conclusion lies in its reliance on correlation analysis, with a lack of mechanistic validation.

An *in vivo* study in a rat model has revealed the negative effect of the NPY-Y1R signaling pathway on the degenerative process [19]. However, research on human lumbar intervertebral discs suggests that NPY, under certain conditions, may exhibit a bidirectional regulatory effect by promoting disc cell proliferation. NPY upregulates the expression of cyclin D1 and proliferating cell nuclear antigen (PCNA), while downregulating the expression of pro-apoptotic proteins such as Bax and caspase-3/8, thereby reducing IL-1β-induced apoptosis [20]. These results indicate that NPY does not exert a solely pro-degenerative effect; instead, its biological impact may depend on the receptor subtype expression and species differences. Additionally, VIP-positive sympathetic nerve fibers have been detected in degenerative rat intervertebral disc tissues [145]. Further studies have shown that the expression of VIP receptors in human intervertebral discs is negatively correlated with the severity of IVDD. Exogenous VIP administration improved IL-1β-induced NPC apoptosis and inflammation. VIP delays the progression of IVDD through the FGF18/FGFR2-mediated Akt signaling pathway [12]; therefore, targeting the VIP signaling pathway may have therapeutic potential for slowing inflammation and apoptosis in IVDD.

In summary, the significant correlation between the expression of ARs, VIP receptors, and NPY in patients with IVDD and clinical parameters supports their potential as biomarkers. However, as a potential therapeutic target for fracture treatment, the safety and efficacy of modulating the ANS still require validation through high-quality clinical trials.

### 4.5 Other musculoskeletal pathologies

Frozen shoulder is characterized by chronic aseptic inflammation and fibrosis of the soft tissues surrounding the shoulder joint, resulting in severe pain and restricted active and passive range of motion [255], [256]. A case-control study revealed that patients with frozen shoulder exhibited significantly greater ANS dysfunction compared to healthy controls [257]. This study directly included human subjects and demonstrated that ANS abnormalities were associated with shoulder tissue fibrosis and pain, indicating significant clinical relevance.

Craniofacial bone defects involve the loss of major facial and cranial bones due to infection, trauma, tumors, or genetic factors [32], [258]. A rat model of mandibular distraction osteogenesis with cervical sympathetic trunk transection demonstrated increased new bone formation following sympathetic denervation, suggesting that the SNS may negatively regulate new bone formation during distraction osteogenesis [259].

Adolescent idiopathic scoliosis (AIS) is a multifactorial three-dimensional spinal deformity that mainly affects children around puberty and is characterized by low body weight, reduced BMI, decreased bone mass, and increased arm span [260], [261]. A Hong Kong, China study of 58 girls with AIS revealed elevated plasma adrenaline and NE levels, which negatively correlated with body weight, BMI, and BMD [260]. These findings suggest that SNS overactivation may drive the “low weight + low bone mass” phenotype of AIS patients, highlighting the potential of SNS inhibition (e.g., β-blockers) to improve bone mass in AIS patients.

Delayed onset muscle soreness is typically observed after unaccustomed or eccentric exercise and is characterized as a transient skeletal muscle condition [262]. Its development is closely associated with mechanical microtrauma, inflammation, and the leakage of enzymes and electrolytes [263], [264]. A randomized controlled trial by Fleckenstein *et al.* [265] confirmed that stellate ganglion block, by temporarily inhibiting the SNS, significantly reduced the expression of inflammation markers such as cell-free DNA and alleviated delayed-onset muscle soreness-related pain.

Bone tumors are categorized into primary and secondary types, with secondary bone tumors referred to as metastatic tumors. Due to its unique activity and regenerative potential, the skeleton has become the third most common site for cancer metastasis, following the lungs and liver [266], [267]. The ANS plays a crucial role in certain symptoms of bone tumors, such as cancer-induced bone pain (CIBP). For example, in a rat model of CIBP, the α7-nAChR agonist PNU-282,987 produced dose-dependent analgesic effects on the spinal cord [266], [267], indicating that spinal α7-nAChR activation is effective for the relief of bone cancer pain.

**Fig. 4:**
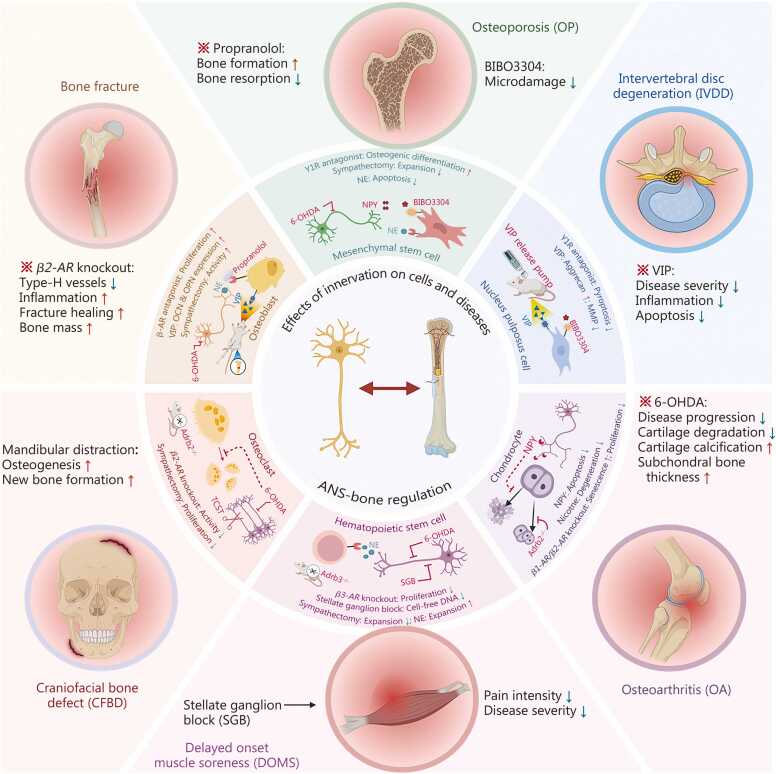
Schematic diagram showing the effects of different types of autonomic nervous interventions or therapies on various bone-related cells and their therapeutic effects on various musculoskeletal disorders. Created with BioRender.com. ↑ Upregulation; ↓ Downregulation; ※ Interventions with significant regulatory impact on the autonomic nervous system. ANS. Autonomic nervous system; Y1R. Neuropeptide Y receptor type 1; NE. Norepinephrine; 6-OHDA. 6-hydroxydopamine; NPY. Neuropeptide Y; VIP. Neuropeptide Y; MMP. Matrix metalloproteinase; OCN. Osteocalcin; OPN. Osteopontin; Adrb. Adrenergic receptor beta; TCST. Transection of cervical sympathetic trunk; DNA. Deoxyribonucleic acid.

Clinical studies suggest that β-blockers can reduce disease-specific and overall mortality in multiple myeloma (MM) patients, suggesting that SNS activation may worsen the prognosis of MM[268], [269]. Some retrospective data also indicate that high expression of cholinergic receptor genes correlated with poor survival outcomes for MM patients [268], [270]. However, increased receptor gene expression does not necessarily imply enhanced downstream biological effects, as receptor expression levels do not always correlate with functional outcomes, underscoring the need for functional validation. AR antagonists, such as propranolol and carvedilol, decrease the viability of both human and canine osteosarcoma cell lines in a dose-dependent manner. Short-term use of these drugs does not significantly affect clonogenic survival, but prolonged low-dose carvedilol treatment increases osteosarcoma cell death after radiation exposure [271]. These findings suggest that carvedilol could be a potential adjunct for radiotherapy in individuals with osteosarcoma, warranting further exploration of its antitumor effects. Ewing sarcoma (ES) has a poor prognosis and is closely linked to metastasis, tumor hypoxia, and chromosomal instability (CIN). A study has shown the hypoxia-induced NPY/Y5R axis stimulates excessive RhoA activation, leading to the formation of polyploid ES cells with high CIN, bone invasiveness, and drug resistance. Blocking Y5R prevents polyploidization and bone metastasis, suggesting that targeting the NPY/Y5R/RhoA axis could inhibit CIN and ES progression [272]. Additionally, persistent expression of NPY and Y5R in ES tumors increases serum peptide levels, which are correlated with poor prognosis. The NPY/Y5R axis drives ES cell motility through RhoA activation, further supporting its critical role in ES metastasis [273]. Breast cancer cells have a strong tendency for bone metastasis, and the SNS facilitates this process by promoting bone resorption. Sympathetic neurotransmitters activate α- and β-ARs, thereby enhancing angiogenesis, immune evasion, and bone metastasis in breast cancer [274]. Campbell *et al*. [275] also indicated that SNS activation (via severe psychological stress and depression) influences the host bone marrow stroma through neurohormones, promoting the colonization of MDA-231 breast cancer cells in mouse bones. Furthermore, propranolol inhibits breast cancer bone metastasis. The upregulation of IL-6 and the JAK/STAT3 signaling pathway may be a key mechanism by which chronic stress promotes bone metastasis in patients with breast cancer, and propranolol effectively inhibits this pathway [276]. Population-based studies also support the use of β-blockers in managing bone metastasis associated with breast cancer [277], and the potential for sympathetic receptor intervention in breast cancer bone metastasis is gradually gaining recognition (Fig. 4, Table 4) [12], [15], [19], [31], [40], [88], [131], [136], [148], [202], [207], [226], [231], [232], [238], [259], [265], [272], [278], [279].

**Table 4:** Therapeutic effects of ANS interventions in bone diseases.

**Disease types**	**Disease models**	**ANS intervention strategies**	**Targets for intervention**	**Bone cell lineage involvement**	**Therapeutic strategies**	**Cell effects**	**Disease effects**	**References**
Osteoporosis (OP)	Tibia fracture models in mice with ovariectomy (OVX)-induced postmenopausal OP	Propranolol (a nonselective β-AR antagonist)	β-AR	Osteoblasts; Osteoclasts	Combined PTH and propranolol	Osteogenesis↑; Osteoclastogenesis↓	OP severity↓; Osteoporotic fracture healing↑	[88]
	Bone microdamage model in rats with OVX-induced postmenopausal OP and *in vivo* fatigue loading	BIBO3304 (a Y1R antagonist)	Y1R	BMSCs	BIBO3304	Osteogenesis, Runx2 & OCN↑; RANKL/OPG ratio↓	OP severity, microdamage↓	[15]
	Femoral fracture models in rats with OVX-induced postmenopausal OP	Chemical SYMX with 6-hydroxydopamine (6-OHDA)	Sympathetic nerves	NA	SYMX	NA	Bone volume↑	[226]
	Rat OVX-induced postmenopausal OP model	Propranolol (a nonselective β-AR antagonist)	β-AR	Osteoblasts	Propranolol	Osteogenesis↑	Mineral density↑; Leptin and leptin receptor level↑→bone formation↑, bone resorption↓	[278]
Osteoarthritis (OA)	Naturally aging mouse model of OA	Propranolol; *β1-AR* knockout, depletion of TH^+^ sympathetic nerves (oxidized dopamine 6-OHDA) or guanethidine)	β-AR; β1-AR; TH^+^ sympathetic nerves	Chondrocytes	Propranolol; *β1-AR* knockout; SYMX	Propranolol: β1-AR and β2-AR↓, Sirt6↑, miR-125↑; *β1-AR* knockout: MMP-13↓, Sirt6↑; SYMX: MMP-13↓	Propranolol: aging-related OA severity↓; *β1-AR* knockout: aging-related OA severity↓; SYMX: aging-related OA↓	[136]
	Monosodium iodoacetate (MIA)-induced knee joint OA mouse model	Nicotine (a nAChR potent agonist); Methyllycaconitine (MLA, α7-nAChR antagonist)	α7-nAChR	Chondrocytes	Nicotine; MLA	Nicotine: aggrecan loss↓, degeneration↓, MMP-9↓; MLA: aggrecan loss↑, degeneration↑, MMP-9↑	Nicotine: OA-induced mechanical allodynia↓, MIA-induced cartilage degradation↓; MLA: OA-induced mechanical allodynia↑, MIA-induced cartilage degradation↑;	[202]
	A mouse model of temporomandibular joint (TMJ)-OA induced by unilateral anterior crossbite	Conditional deletion of β2-AR in the nestin + MSCs	β2-AR	MSCs; Chondrocytes; Osteoclasts	Conditional deletion of β2-AR in MSCs	Osteoclast: hyperfunction↓; Chondrocytes: cartilage thickness, cell density, proteoglycan-positive area, collagen II, aggrecan, *Col2a1* & *aggrecan* mRNA↑; MMP-13, MMP-3, calcified cartilage, ALP-positive area, *ALP* mRNA↓	Cartilage degradation, calcified cartilage thickening, subchondral bone loss↓	[279]
	The OA model of wild-type and SYMX mice induced by destabilization of the medial meniscus (DMM)	SYMX by intraperitoneal injections of 6-OHDA	TH^+^ sympathetic nerves	Chondrocytes; Osteoblasts; Osteoclasts	SYMX	Chondrocytes: no influence on β2-AR; Osteoblast: activity↑; Osteoclast: activity↓	Cartilage calcification, subchondral bone thickening↑	[131], [207]
	The right knee OA model of 8-week-old male mice induced by DMM	Intra-articular injection of NPY	NPY1R; NPY2R	Chondrocytes	BIBO3304; BIIE0246	BIIE0246: Col10a1↓, MMP-13↓, ADAMTS-5↓, pS6-positive chondrocytes↓, apoptosis↓, hypertrophy↓	BIIE0246: NPY-induced cartilage degradation and fibrillation↓; BIBO3304: no effect on NPY-induced cartilage degradation and fibrillation	[31]
Bone fracture	Tibia fracture mouse model of chronic posttraumatic pain with features of complex regional pain syndrome	Chemical SYMX by intraperitoneal injections of 6-OHDA; Inhibition of sympathetic outflow with Lofexidine (an α2-AR agonist); Parasympathetic augmentation with nicotine	TH+sympathetic nerves; α2-AR; nAChR	NA	SYMX; Lofexidine; Nicotine	NA	SYMX, nicotine, lofexidine: fracture-induced nociceptive sensitization and inflammation↓	[232]
	Murine femoral osteotomy combined with cortical impact brain injury/traumatic brain injury (TBI) model	A hyperadrenergic state induced by TBI	Sympathetic nerves; β2-AR	Osteoblasts; Osteoclasts; Periosteal cells	*β2-AR* knockout; propranolol; atenolol; formoterol	*β2-AR* knockout: osteogenesis↑, osteoclastogenesis↓; *β2-AR* knockout: VEGF-A↓ in periosteal cells, hypoxia-inducible factor 1α↓ in periosteal cells;	*β2-AR* knockout: bone mass in the unfractured skeleton↑, fracture healing↓; Propranolol & formoterol: fracture nonunion↑, type-H vessels density ↓; Atenolol: no effect on fracture	[231]
	Mouse TBI combined with the femur fracture model	Chemical SYMX with 6-OHDA; *β2-AR* and *β3-AR* knockout	Sympathetic nerves; β2-AR; β3-AR	BMSCs; BHSCs; BMDMs	SYMX; *β2-AR* and *β3-AR* knockout; NE; Clenbutero l; BRL37344; Combined clenbuterol & BRL37344	SYMX: proliferation ↓ in BMSCs & BHSCs; SYMX: OCN, OSX, OCPs, TRAP^+^ cells, anti-inflammation myeloid cells↓; NE: expansion ↑ in BHSCs; *β2-AR* knockout: M2 polarization ↓ in BMDMs; *β3-AR* knockout: proliferation ↓ in BHSCs; β-AR agonist: M2 macrophages↑, OCN↑	SYMX: TBI-accelerated fracture healing↓; β-AR agonist: fracture healing ↑	[148]
	Mouse closed tibial fracture model	Germline *Y1R* knockout; Osteoblastic-specific *Y1R* knockout	Y1R	Chondrocytes; Osteoblasts	Germline *Y1R* knockout; Osteoblastic-specific Y1R knockout	Germline *Y1R* knockout: chondrocyte hypertrophy↑	Germline *Y1R* knockout: cartilage callus removal↓, hard callus formation↓→ early fracture healing↓; Osteoblastic-specific *Y1R* knockout: no effect on fracture healing	[238]
	Mouse femur transverse fracture model	Chemical SYMX with 6-OHDA	Sympathetic nerves	Osteoblasts	VIP	OCN, OPN↑	SYMX-impaired bone fracture healing↑	[40]
Intervertebral disc degeneration (IVDD)	Rat IVDD model established by percutaneous disc puncture	Needle puncture induces aberrant distribution of NPY in the degenerative disc	Sympathetic nerves	Nucleus pulposus cells (NPCs)	BIBO3304	Pyroptosis↓	Pyroptosis↓→ IVDD development↓	[19]
	Mice lumbar instability-induced lumbar IVDD model	The injection of exogenous VIP into mice with IVDD via a sustained-release pump	VIP2R	NPCs	VIP	ACAN↑, MMP-3↓	Lumbar instability-induced IVDD development↓	[12]
Craniofacial bone defect (CFBD)	Rat bilateral transection of cervical sympathetic trunk (TCST), mandibular distraction osteogenesis model	Bilateral TCST	Sympathetic nerves	Osteoclasts	Transection of the sympathetic innervation	Bone mass, quantity of trabeculae ↑	New bone callus maturity↑	[259]
Delayed onset muscle soreness (DOMS)	DOMS of the biceps muscle model	Stellate ganglion block (SGB)	Sympathetic nerves	Hematopoietic cells	SGB before exercise-induced DOMS (preventive); SGB after the induction of DOMS (rehabilitative)	cell-free DNA↓	DOMS severity↓, DOMS-related pain intensity↓	[265]
Ewing sarcoma	Orthotopic Ewing sarcoma xenograft model	Blocking Y5R	Y5R	SK-ES-1 cell line (human Ewing sarcoma cell line)	CRISPR/Cas9 *NPY5R* gene editing	Polyploid cell formation, chromosomalinstability↓, and genomic stability of tumor cells↑	Bone metastasis, chemoresistance, disease progression↓	[272]

↑ Upregulation; ↓ Downregulation. NA. Not applicable; ANS. Autonomic nervous system; AR. Adrenergic receptor; PTH. Parathyroid hormone; Y1R. Neuropeptide Y receptor type 1; BMSCs. Bone marrow mesenchymal stem cells; Runx2. Runt-related transcription factor 2; MSCs. Mesenchymal stem cells; OCN. Osteocalcin; RANKL. Receptor activator of nuclear factor kappa-B ligand; OPG. Osteoprotegerin; SYMX. Sympathectomy; MMP. Matrix metalloproteinase; nAChR. Nicotinic acetylcholine receptor; MLA. Methyllycaconitine; mRNA. Messenger ribonucleic acid; ALP. Alkaline phosphatase; NPY. Neuropeptide Y; NE. Norepinephrine; BHSCs. Bone marrow hematopoietic stem cells; BMDMs. Bone marrow-derived macrophages; VIP. Vasoactive intestinal peptide; TH. Tyrosine hydroxylase; Sirt6. Sirtuin 6; OSX. Osterix; OCPs. Osteoclast precursors; TRAP. Tartrate-resistant acid phosphatase; Col10a1. Collagen type X alpha 1 chain; ADAMTS-5. A disintegrin and metalloproteinase with thrombospondin motifs 5; VEGF-A. Vascular endothelial growth factor A; ACAN. Aggrecan; NPY5R. Neuropeptide Y receptor Y5

## 5 Conclusion and future perspective

The ANS, through its sympathetic and parasympathetic branches, exerts regulatory control over various cell types [8], [94], particularly those associated with bone, joints, and intervertebral discs, including MSCs, osteoblasts, osteoclasts, NPCs, and chondrocytes. By releasing neurotransmitters such as NE, ACh, NPY, and VIP, the ANS modulates cellular behaviors including proliferation, differentiation, migration, and functional activity, thereby influencing the development, metabolism, remodeling, and repair processes of skeletal tissues. In the context of bone-related disorders, dysfunction of the SNS and PSNS has frequently been linked to dysregulated bone metabolism, as observed in conditions such as OP, OA, traumatic fractures, and IVDD. This correlation appeared especially pronounced in age-related skeletal pathologies, highlighting an urgent need to elucidate the complex interplay between autonomic inputs, neurotransmitter signaling, and downstream cellular responses. With growing insights into the role of the ANS in bone homeostasis, neuromodulation-based strategies have emerged as promising therapeutic approaches for bone-related disorders.

Recent advances in both neuromodulatory devices and pharmacological interventions have demonstrated substantial potential. By finely tuning the activity of the SNS and PSNS, these strategies effectively modulated bone metabolism and enhanced bone repair and regeneration. For instance, VNS was reported to slow the progression of OA [215]. Pharmacological interventions, particularly highly selective β1-AR antagonists, hold substantial translational promise for osteoporotic neuromodulation [88], [108], [280]. Nevertheless, off-target cardiovascular effects and suboptimal delivery efficiency continue to hinder their broader clinical application. Future therapeutic strategies should combine these sympathetic inhibitors with bone-targeted nanocarriers to maximize osteogenic benefits while circumventing systemic toxicity. Moreover, tVNS has emerged as a promising non-invasive strategy for OA [216]. Compared with systemic central analgesics (such as SNRIs) [214] and irreversible nerve ablation [131], [207], tVNS offers a safer alternative. Mechanistically, it mitigates central pain sensitization via the cholinergic anti-inflammatory pathway while concurrently suppressing synovial inflammation and delaying cartilage degeneration. However, conventional fixed-stimulation protocols are hindered by unstandardized parameters and substantial inter-individual variability in baseline autonomic tone. Future strategies should focus on personalized, feedback-driven systems. By integrating objective biomarkers such as heart rate variability to dynamically adjust stimulation, this adaptive approach can optimize analgesic and joint-protective outcomes across diverse OA cohorts.

For fractures and IVDD, the localized delivery of autonomic neuromodulators (such as VIP or NPY-Y1R antagonists) via smart biomaterials, including stimuli-responsive hydrogels [281], [282], offers a promising therapeutic approach. By directly remodeling the local microenvironment, this method prevents the off-target effects of systemic delivery, such as unintended bone loss. During fracture repair, this intervention enhances callus stability while maintaining systemic homeostasis. In degenerated discs, it interrupts the inflammatory cascade driven by abnormal sympathetic ingrowth, inhibits apoptosis, and reverses ECM degradation to drive structural repair. However, clinical translation of this approach faces two primary challenges. First, the hostile intradiscal environment, including hypoxia, acidity, and elevated mechanical stress, restricts the prolonged release of these therapeutic agents [283], [284], [285]. Second, the high spatiotemporal heterogeneity of neural signaling during fracture healing complicates the precise control of the therapeutic window [286]. Future research should develop spatiotemporally controlled delivery systems that respond to local microenvironmental changes for on-demand drug release. Advances in precision medicine are expected to facilitate the development of personalized neuroregulatory therapies and promote multidisciplinary collaboration, thereby accelerating the clinical translation of nervous system-based treatments for bone diseases.

Although the role of the ANS in bone metabolism has garnered significant attention, several key challenges hinder the integration of mechanistic understanding and its clinical translation. First, accumulating evidence indicates that ANS signaling is highly context-dependent. Biological responses can vary considerably based on factors such as receptor subtype (e.g., β2-AR/β3-AR, D1/D2), ligand concentration, stimulation duration, or the pathological state of the cells. For example, activation of ARs suppresses both osteoclastogenesis and osteogenic differentiation of MSCs [82], [114], while the effects of ACh are concentration-dependent [287], [288]. Additionally, neurotransmitter interactions *in vivo*, influenced by receptor subtypes and the local microenvironment, further complicate interpretation and often lead to inconsistent results. Second, the translation of animal findings into clinically actionable strategies remains limited. Significant physiological differences between animals and humans raise concerns regarding the direct applicability of preclinical results [289]. Importantly, ANS modulation is inherently systemic, and prolonged pharmacological or neuromodulatory interventions may influence cardiovascular and metabolic functions [290], [291]. Third, disease-specific neural signatures have been proposed, with sympathetic overactivity being associated with low body weight, reduced bone mass, and abnormal bone metabolism in AIS [260], as well as with metabolic alterations in OP [187], [188] and breast cancer bone metastases [277]. Reliable screening markers, such as receptor expression levels or sensitivity indicators, remain unavailable, contributing to substantial inter-individual variability in clinical outcomes.

Therefore, future studies should clarify the subtype-specific mechanisms of different autonomic branches and establish reliable biomarkers to accurately assess neural status, which will be essential for the precise clinical translation of neuromodulatory strategies.

## Abbreviations

2-AG: 2-Arachidonoylglycerol

α-ARs: Alpha-adrenergic receptors

β-ARs: Beta-adrenergic receptors

ACh: Acetylcholine

Adrb2: Adrenergic receptor beta 2

AIS: Adolescent idiopathic scoliosis

Akt: Protein kinase B

ALP: Alkaline phosphatase

ANS: Autonomic nervous system

AP-1: Activator protein 1

ARs: Adrenergic receptors

ATF4: Activating transcription factor 4

BHSCs: Bone marrow hematopoietic stem cells

BMAT: Bone marrow adipose tissue

BMD: Bone mineral density

BMI: Body mass index

BMSCs: Bone marrow-derived mesenchymal stem cells

cAMP: Cyclic adenosine monophosphate

CB1: Cannabinoid receptor type 1

CB2: Cannabinoid receptor type 2

CIN: Chromosomal instability

CNS: Central nervous system

CTX: C-terminal telopeptide of type I collagen

DA: Dopamine

D1R: Dopamine receptor D1

ECM: Extracellular matrix

ECS: Endocannabinoid system

ERK: Extracellular signal-regulated kinase

ES: Ewing sarcoma

GPCR: G protein-coupled receptor

HSCs: Hematopoietic stem cells

HSPCs: Hematopoietic stem and progenitor cells

IL: Interleukin

IVDD: Intervertebral disc degeneration

M3R: Muscarinic acetylcholine receptor M3

mAChRs: Muscarinic acetylcholine receptors

MMP: Matrix metalloproteinase

MSCs: Mesenchymal stem cells

nAChRs: Nicotinic acetylcholine receptors

NE: Norepinephrine

NFATc1: Nuclear factor of activated T cells cytoplasmic 1

NPCs: Nucleus pulposus cells

NPY: Neuropeptide Y

NPY2R: Neuropeptide Y receptor Y2

OA: Osteoarthritis

OCN: Osteocalcin

OP: Osteoporosis

OPG: Osteoprotegerin

OPN: Osteopontin

Pdcd4: Programmed cell death 4

PKA: Protein kinase A

PSNS: Parasympathetic nervous system

PTH: Parathyroid hormone

RA: Rheumatoid arthritis

RANKL: Receptor activator of nuclear factor kappa-B ligand

ROS: Reactive oxygen species

SNRI: Serotonin and norepinephrine reuptake inhibitor

SNS: Sympathetic nervous system

SOX9: SRY-related HMG-box transcription factor 9

SYMX: Sympathectomy

TBI: Traumatic brain injury

TNF: Tumor necrosis factor

TRAP: Tartrate-resistant acid phosphatase

tVNS: Transcutaneous auricular vagus nerve stimulation

VIP: Vasoactive intestinal peptide

VNS: Vagus nerve stimulation

VPAC: Vasoactive intestinal peptide receptor

## Ethics approval and consent to participate

Not applicable.

## Authors’ contributions

YSL and WFX conceived the study and revised the manuscript. WJH and YKH drafted the manuscript and contributed to the viewpoint discussions. HZL, WHL, GY, and BZJ assisted with figure preparation and revision discussions. All authors read and approved the final manuscript.

## Funding

This work was supported by the National Key R&D Program of China (2023YFC3603400), the National Natural Science Foundation of China (82472522, 92268115), the Projects of International Cooperation and Exchanges NSFC (W2421123), and the Hunan Provincial Science Fund for Distinguished Young Scholars (S2024JJJCQN0405).

## Competing interests

The authors declare that they have no competing interests.

## Data Availability

Not applicable.
